# Bacterial Cellulose and Its Applications

**DOI:** 10.3390/polym14061080

**Published:** 2022-03-08

**Authors:** Soon Mo Choi, Kummara Madhusudana Rao, Sun Mi Zo, Eun Joo Shin, Sung Soo Han

**Affiliations:** 1 Research Institute of Cell Culture, Yeung-Nam University, Gyengsan-si 38541, Korea; smchoi@ynu.ac.kr; 2 School of Chemical Engineering, Yeung-Nam University, Gyengsan-si 38541, Korea; msraochem@yu.ac.kr (K.M.R.); sunmizo@ynu.ac.kr (S.M.Z.); 3 Department of Organic Materials and Polymer Engineering, Dong-A University, Busan 49315, Korea

**Keywords:** bacterial cellulose, biocellulose, microbial cellulose, biodegradable cellulose

## Abstract

The sharp increase in the use of cellulose seems to be in increasing demand in wood; much more research related to sustainable or alternative materials is necessary as a lot of the arable land and natural resources use is unsustainable. In accordance, attention has focused on bacterial cellulose as a new functional material. It possesses a three-dimensional, gelatinous structure consisting of cellulose with mechanical and thermal properties. Moreover, while a plant-originated cellulose is composed of cellulose, hemi-cellulose, and lignin, bacterial cellulose attributable to the composition of a pure cellulose nanofiber mesh spun is not necessary in the elimination of other components. Moreover, due to its hydrophilic nature caused by binding water, consequently being a hydrogel as well as biocompatibility, it has only not only used in medical fields including artificial skin, cartilage, vessel, and wound dressing, but also in delivery; some products have even been commercialized. In addition, it is widely used in various technologies including food, paper, textile, electronic and electrical applications, and is being considered as a highly versatile green material with tremendous potential. However, many efforts have been conducted for the evolution of novel and sophisticated materials with environmental affinity, which accompany the empowerment and enhancement of specific properties. In this review article, we summarized only industry and research status regarding BC and contemplated its potential in the use of BC.

## 1. Introduction

Recently, effort has made a streak for green technologies where the sustainable economy brings innovation and novel paradigms by reducing petroleum-based materials consumption and their alternative with recyclable and circular products [1]. Among all-natural materials, cellulose is one of the most abundant biodegradable biomasses on the earth, which is a polysaccharide composed of a linear chain of β-1, 4 linked d-glucose unit. Cellulose is produced by a number of living organisms ranging from bacteria to plants, which are obtained through top-down and bottom-up approaches, respectively [2,3,4]. 

In addition to plant-derived cellulose obtained from wood, cotton, and bamboo, cellulose can also be synthesized by various species of bacteria, including the genera *Acetobacter*, *Gluconobacter*, *Komagataeibacter*, *Rhizobium*, *Agrobacterium*, and *Sarcina*; this is referred to as bacterial cellulose (BC) or microbial cellulose (MC). The BC has a nanostructure which determines its physical and mechanical properties, and its shaping can be controlled by the type of cultivation and bioreactor and then its obtained hydrogel or dry state by freeze-drying. Optimal medium design is very important for the growth of microorganisms, which respond to changes in their environment in many ways, such as the induction and inhibition of protein synthesis, and changes in cell morphology. Compared with plant cellulose, BC, being thinner, and a nanofiber weaved 3D network allows to increase the surface-area-to-volume ratio, enabling strong interaction with neighboring components [5]. It also provides relatively outstanding mechanical strength, a high degree of polymerization and crystallinity (about 90%), and water holding capacity. Additionally, it is gaining attention owing to its purity (free lignin, hemicellulose, pectin, and etc.), no toxicity, and biodegradability. These properties allow its applications in the medical field as scaffolds with wound healing properties for tissue regeneration. Moreover, it can be hybridized or grafted chemically with various biopolymers by several hydroxyl functional groups throughout the chain, allowing to be functionalized and employed in the fabrication of nanocomposites [6].

BC properties such as tensile strength, degree of polymerization, crystallinity, and moisture absorbing can be altered depending on several factors including culturing conditions, the kind of microorganism, and nutrients comprised in the growth medium. Studies have shown the effect of dehydration/rehydration of BC on its tensile strength [7], the influence of a polymer treatment method on mechanical properties [8], the effect of the microorganism type and drying method on Young’s modulus value [9], and the effect of the composition and pH of the culture medium on crystallinity [10]. Additionally, it was established that the impregnation of alginate in BC allowed to improve its water retention [11], and it was found that the addition of beeswax into BC film was possible to increase its hydrophobicity by confirming its contact angle [12]. It was also confirmed that the incorporation of collagen between bacterial cellulosic fibers allowed the fabricated BC-based materials to enhance thermal stability as well as its cytocompatibility by collagen fibrillogenesis [13].

The advantages mentioned above make BC an ideal candidate for sustainable sources of cellulose biomaterials; accordingly, it has been researched and also commercially used in numerous applications. This crucial review article establishes the progress, especially focusing on an up-to-date development, in detail, of the commercial and researchable applications including food, biomedical, paper, textiles, electrical and electronic industry, etc. Finally, the future prospects of BC are summarized. 

## 2. Applications

### 2.1. Food Industry

Being a dietary fiber, BC has been considered ‘‘generally recognized as safe’’ (GRAS) by the Food and Drug Administration (FDA) since 1992 [14,15]. BC possesses manifold potentialities in food industries owing to its high purity; variety of textures and shapes (e.g., particles, spheres, filaments, multi-shaped pulps, films, and whiskers); capability to acquire in situ changes, such as colors and flavors of culture medium; and easy production process [14]. Considering the above-mentioned properties, BC can be used as an adjuvant in foods and the food industry.

#### 2.1.1. Traditional Dessert

BC can be utilized in various disciplines due to its gel formation, emulsifying, and water maintenance. For example, the addition of BC to food provides firmness, retention properties, and better texture. The incorporation of a specific taste and flavor may also improve the quality of certain foods. For a long time, it has been employed as the raw material nata de coco, an indigenous dessert food of the Philippines, for which one-centimeter-thick gel sheets fermented with coconut water are cut into cubes and immersed in sugar syrup. The nata de coco is utilized as a sweet candy dessert. It has converted into a very well-linked food and is now quickly spreading worldwide in a dessert or candy [16]. Similar food can be prepared from other saps or fruit juices, e.g., nata de pina from pineapple. The major component of nata de coco gel is cellulose, as proved in 1960s [17]. Nata de coco is now manufactured in a large quantity at the level of home industry in Indonesia and is exported as a healthy food. Teekvass, or tea fungus, grown in teacups and served in some parts of Russia and Middle Asia, is said to be a similar ferment [2]. In South Korea, it has been imported and used in the Philippines and Thailand, but Zadam Co., Ltd. has been involved in research on the production of BC, a high-value functional food, since May 2012. Based on BC production technology transferred from Jeju Gamgyul Testing Center of the Rural Development administration in March 2011, it replaces some of the coconut cellulose imported in the total quantity. In addition, research is underway to develop antiobesity efficacy materials using Gamgyul (a kind of mandarin) biocellulose [18].

#### 2.1.2. Low Calorie and Low Cholesterol Products

For almost two decades, the use of BC as non-caloric materials was evaluated due to its water-holding capacity and smooth texture. As BC has a higher water uptake and cation exchange capacity, lipid blood, and cholesterol-lowering effect than plant-based cellulose, it can be utilized in food additives such as fat-free, low-cholesterol, and low-calorie foods [19]. The employment of BC to oily food products, for example, margarine or mayonnaise, was not viable because its texture does not resemble cream [20]. Hence, its use for has been considered for low-fat meat products, such as hamburger patties, sausage, and as a fat replacer in emulsified meat products [14,15,21]. The reduction in calories was pointed at 25% and in accordance to the recommended low-calorie labeling standards of the U.S. Department of Agriculture (USDA) [17]. The calories of the patty dropped from 258 kcal to 194 kcal per 100 g with no impact on the mildness and juiciness by substituting BC and beef extract for a third of beef. The fat amount of the patty using BC was 10%, and it satisfied the standards of a ‘low-fat hamburger’ as recommended by the USDA [2]. Similar effects on reducing calories were obtained in sausage. The soy-protein-based sausage consisted of a rough water dispersion, lard, pork, and soy protein, while the BC-based sausage partially included BC paste. Despite being a low-fat configuration, stable emulsions were manufactured by adding BC. The result showed that the BC-based sausage with low fat of 12% was obtained, which reduced the calories by about 25%. It was considered that the addition of BC did not have a decisive effect on the sausage’s texture, as the low-fat ground sausage was quite uneven and fragile [22]. Lin and Lin evaluated the quality characteristics of Chinese meatballs with 20% fat according to various amounts of BC (Nata) in 2004. It was exhibited that the addition of 10% BC to Chinese meatballs not only has a sufficient texture and shelf stability but also sensory qualities with no effect on texture. Moreover, it offered suitable juiciness and chewiness to foods, making BC a potential food additive in emulsified meat products, including Chinese meatballs. Lin et al. examined the addition of BC with a high water-uptake ability to dolphinfish (mahi-mahi) surimi. An astonishing reduction in rigidity as well as gel strength of heated surimi was found when the alkali-treated BC of 5% was added. The surimi–BC exhibited an excellent water-holding capacity, caused by the network of alkali-treated BC [23]. Marchetti et al. discussed the use of BC to low-lipid and low-sodium meat emulsions composed of pre-emulsified high-oleic sunflower oil in 2017. The high hydrophilicity and surface area of BC brought about various results to improve the water-binding property, increased the process yield, water-holding capacity, and enhanced texture similar to be commercial, resulting in the potential to stabilize meat products and be a fat replacement for beef [24]. Akoglu et al. studied the effects of BC on fat-reduced mayonnaise. There was no difference (*p* > 0.05) between the samples including control (0%), M1 (0.25%), M2 (0.5%), M3 (0.75%), M4 (1.0%), and M5 (2.0%), labelled according to the content (%) of BC, in sensory properties such as smell, thickness, oiliness, and aftertaste, of fat-reduced mayonnaise [25]. The effect of BC on the texture, thermal stability, emulsifying properties of BC-based soy protein isolate (SPI) complex gel as fat replacer in ice cream was investigated by Guo et al. in 2018 [26]. The neat SPI was not stable at room temperature, owing to protein aggregation without BC. In contrast, the BC-based suspension was remarkably enhanced in terms of the stability of the BC–SPI mixtures, suggesting that it could be an additive for stabilizing emulsions. They supposed the mechanisms; firstly, both BC and SPI absorbed on the surface of oil droplets and disturbed the aggregation of the droplets. Secondly, the BC and SPI, which were not absorbed, interacted through non-covalent bonds to generate a gel-like network. Therefore, the emulsion stability was enhanced by disturbing the oil-droplet aggregation. Consequently, the addition of BC allows to prevent the phase separation and to improve the emulsion stability. BC can be included in ice cream to decrease its calories and enhance the melting resistance and textural properties. 

Since BC has a higher water uptake and cation exchange capacity, lipid blood, and cholesterol-lowering effect compared to plant-based cellulose, it can be utilized in the field such as fat-free, low-cholesterol, and low-calorie foods [27]. Recently, it has been studied as a potential candidate as a fat-substitute material in emulsified meat products. It was found that BC lowered the cholesterol content in an in vitro test by absorbing or binding it in the body [28]. BC gel is as hard as bone and has the texture of mollusks such as squid; however, when manufactured with alginate and calcium chloride or sugar alcohol to increase the water content, a chewable texture can be obtained. These features make it possible to use it in new food for low-calorie desserts or salads [29]. 

#### 2.1.3. Food Additive and Dietary Aid

In the past decade, BC has been evaluated for its availability as a stabilizer, suspension, thickening, and gelling agent in the food industry, and has also played a role in a heat-stable suspending agent, and as a reinforcing material for fragile food hydrogel to improve the value of pasty food by reducing its stickiness. For example, the addition of 0.2~0.3% in BC allows the texture of tofu, a food with a soft cheese-like consistency made from soya-bean milk, to be enhanced by increasing the gel strength of the tofu. Adding it to Kamaboko, processed Japanese seafood products made from surimi, improves its rigidity and brittleness enough to tolerate the aging process. Correspondingly, as BC is produced in an agitated culture medium added into a chocolate drink, the precipitation of the cocoa is prevented by BC mesh, which has excellent thermal stability; the viscosity remains after autoclaving [30]. Furthermore, the stickiness could be significantly improved by adding BC into a creamy sauce, making it easier to spoon the proper amount [27]. As above, it has been evaluated as a food additive for improving the stability across a wide range of temperatures, pH values, and freezing/thawing conditions; from this, it is clear that it could be applied as a potential food additive without the restriction of the calories of the mixture products. 

#### 2.1.4. Food Packaging Materials

A large amount of waste is produced every year due to the consumption of packages, which has raised environmental concerns. The materials related to food packaging materials involve polyethylene, polypropylene, polystyrene, polyvinyl chloride, and polyethylene terephthalate, accounting for about 90% of plastic production. These materials have the advantage of being inexpensive; however, they have the disadvantage of not being biodegradable. 

In response to the environmental issues induced from the above-mentioned plastics, sustainable packaging materials are in demand. Recently, biodegradable films have been developed from renewable materials, but they cannot yet completely substitute plastics. Among many potential candidates, BC is drawing attention for use in upcoming alternative materials with special characteristics, which differentiate from other polysaccharide-based polymers.

BC may be a suitable alternative as food packaging material in order to store foods and extend their expiration dates, due to a porous, thin reticulated structure, resulting in it filtering dust, microorganisms, and fungus presented in the air. BC formed by the Gluconacetobacter hansenii HE1 strain was determined comparing with petrochemical materials for the extension of food shelf-life through the colony counts method for the microbial load. It was confirmed that sausage wrapped with BC films could filtrate airborne contamination such as microbes and fungi, which was found to be 1.2 × 10^4^ cfu/mL, better than 2.7 × 10^5^ cfu/mL in one wrapped with petrochemical-derived cling wrap and 1.0 × 10^6^ cfu/mL in an unwrapped one [31]. Additionally, oxidized BC-based composite materials with PVA–chitosan nanofibers were fabricated via needleless electrospinning, which showed their mechanical properties and the water vapor transmission rate, indicating conformity for food packaging materials [32].

There are studies using bacterial cellulose nanocrystals (BCNC) by isolating their crystalline regions for use as reinforcing materials in films for food packaging. Salari et al. developed BCNC-based nanocomposite films with silver nanoparticles (AgNPs) and a chitosan dispersion. It was confirmed that the antibacterial activity, barrier, and tensile properties of the fabricated films was enhanced by the hydrogen bonds formed between chitosan and BCNC [33]. It was also established that BCNC was employed as a reinforcing filler for corn starch films [34] and that nanocomposite PVA films loaded with 15 wt% of BCNC showed enhanced properties including thermal stability, tensile strength, and elastic modulus compared to pure PVA [35]. Additionally, it was reported by Ilyas et al. that a tapioca-starch–chitosan-based film complexed with bacterial cellulose nanofiber improved its tensile strength and thermal resistance [36].

### 2.2. Biomedical Industry

The increasing demand for bio-based BC materials is gaining more attention for immediate applications in biomedical fields such as wound healing, tissue engineering, and drug delivery. BC promotes re-epithelization by stimulating cellular adhesion, proliferation, migration, and differentiation, leading to faster wound healing for regenerative medicine. BC can also be used in biomedical devices such as dental implants, artificial blood vessels, and vascular grafts, as well as in urethral, nerve, artificial cornea, and retina implants as it has key characteristics such as non-toxicity; the ability to maintain moist at the wound site, allowing the exchange of gaseous molecules; low tissue adhesion; and thermal insulation. Moreover, the interfacial attachment between cells and BC plays a crucial role in its performance for in vivo implantation. In addition to biological performance, the BC shows excellent physicochemical and pharmacological properties because of the surface charge, topography, and wettability. Recently, several reviews have been summarized based on BC materials for biomedical applications [37,38,39]. However, insight into the potential applications of BC from a biomedical point of view is still limited. Herein, we report the insights focused on BC products for biomedical applications.

#### 2.2.1. Wound Dressings

Wound healing is a complex process involving four phases: hemostasis, inflammatory, proliferation, and maturation. The promotion and functioning of these phases depend on the wound type, pathological conditions, and dressing material [40]. An ideal wound dressing should maintain a moist environment, absorb exudates, support angiogenesis, allow gaseous exchange, create thermal insulation in the wound area, and prevent microbial infections. It should have characteristics such as being non-toxic, non-adherent, and non-allergenic, and should be easy to remove without trauma and made with minimal processing and cost-effective material wound healing such as re-epithelialization, autolytic debridement granulation tissue formation, and inflammation [41].

So far, various wound dressings have been developed using natural polymers such as cellulose, alginate, chitin, chitosan, collagen, gelatin, fibrin, etc. Bacterial cellulose (BC) has attractive features in wound healing, including its suitability as a wound-dressing material, elasticity, bacterial inhibition, vapor permeability, and low cost. Owing to the presence of –OH groups and nanofibril networks present in the BC, it is possible to capture water molecules easily by –OH groups and the nanofibril network, which can mimic the skin surface, providing a good environment for wound healing. BC has already been successfully used in wound-care applications, highlighting its potential as a high-value material in healthcare and biotechnology. BC has demonstrated that, through its unique properties, it can meet all of the expectations that a 21st-century wound-care product requires. 

There are several commercial BC products available such as BioFill^TM^ (Curitiba, Brazil), Dermafill^TM^ (Londrina, Brazil), Membracel^®^ (Curitiba, Brazil), xCell^®^ (New York, NY, USA), Gengiflex^®^ (Curitiba, Brazil), and Bioprocess^®^ (Curitiba, Brazil). Companies such as BioFill^TM^ and Dermafill^TM^ have used BC for wound dressings to treat burns and ulcers on patients, which provide the immediate cessation of some pain and discomfort. BioFill^®^ dressings showed all the combined wound-healing properties for effective wound-healing in 300 test cases [42]. Another important wound-dressing material is never-dried BC, which offers fast absorption capability of wound exudates, leading to faster wound-healing. The resulting BC wound dressings (Dermafill^TM^) are further applied to chronic injuries that can improve the epithelization (75%) in 81 days as compared to without BC (315 days) [43]. Vuelo Pharma developed another valuable product, Membracel^®^, for venous leg ulcers and lacerations.

Similarly, the xCell^®^ product was designed by Xylos Corporation for venous leg ulcers. The antimicrobial BC product incorporated with silver ions (Nanoskin^®^ (São Carlos, Brazil)) was developed by Innovatec, which provide an antibacterial property for wound dressing. EpiProtect^®^ (Royal Wootton Bassett, UK) is a wound dressing for burn wounds, which provides complete healing at day 28 for first- and second-stage burns compared to silver sulphadiazine and gauze, which provide complete healing at day 32 [44]. 

Although BC acts as a physical barrier and possesses unique physicochemical properties in wound healing, BC does not meet all of the requirements of the current wound-dressing market. The biofunctionalization of BC loaded with antibacterial drugs and other antimicrobial agents is more beneficial as they kill bacteria around the wound during wound healing. Moreover, the combination of these materials further shows the synergy property to induce faster tissue regeneration of severe injuries. 

Chitosan (CS) is one of the most important biopolymers for wound dressings. Incorporating CS into BC could promote the massive absorption of water compared to BC alone [45,46,47]. Moreover, the degradation of CS into N-acetyl-beta-D-glucosamine further promotes the fibroblast growth and deposition of collagen. A ciprofloxacin (Cip)-loaded CS–BC patch was developed by Cacicedo et al. A BC–Chi–Cip patch was cytocompatible with human fibroblasts [45]. The patch released Cip with a 30% and CS displayed synergy antibacterial activity against *Pseudomonas aeruginosa* and *Staphylococcus aureus* for potentiality wound healing. Furthermore, incorporating glycerin as a plasticizer into BC provides a moisturizing effect on burn wounds [48].

Hydrogels have been extensively used in wound-dressing applications as they can offer a moist environment, which favors re-epithelization. Mohamad et al. developed a BC–acrylic-acid (AA) (BC–AA) hydrogel by electron-beam irradiation for burn wound dressings [49]. The hydrogel had a massive water-absorption capability (4000–6000% within 24 h) and high water-vapor transmittance (WVTR) (2175–2280 g/m^2^/day. The in vivo study proved that the hydrogel promotes fast wound-healing, re-epithelization, and fibroblast growth compared to BC alone. The addition of human epidermal keratinocytes and dermal fibroblasts into BC–AA hydrogel further accelerates burn wound healing [50]. The wound reduction significantly increased for cells loaded hydrogel (77.34 ± 6.21%) on day 13 than BC–AA hydrogel without cells (64–79 ± 6.84%). Moreover, the mouse-treated hydrogel loaded with cells further improved collagen deposition, indicating that the hydrogel is promising for wound dressing and cell carrier.

Furthermore, incorporating silver nanoparticles (AgNPs) impregnated into the BC matrix accelerates wound healing. The AgNPs have been extensively studied for antimicrobial properties. The researchers believe that the silver ions had antibacterial activity against various pathogens via breaking of the cell membrane and DNA replication and transcription. In addition to this, the AgNPs can also promote the release of reactive oxygen species (ROS) and kill bacteria [51]. The silver nanoparticles (AgNPs)-impregnated composites are transparent, which allows the visualization of the progress of wound healing at the wound site. Pal et al. developed silver-functionalized BC for potential wound healing applications. A facile green method was used to prepare AgNPs impregnated into BC matrix by UV-light irradiation treatment. The composite matrix showed good antibacterial activity against Gram-negative *Escherichia coli* (*E.coli*) bacteria [52]. Jiji et al. developed in situ impregnating AgNPs in BC-matrix-coated polydopamine (PDA) via redox catechol chemistry for third-degree burn wound healing [53]. Due to catechol moieties in PDA coated on BC, it can quickly reduce the Ag ions to AgNPs via the redox chemistry of catechol to quinone conversion. The appearance of digital photographs represents a color change of BC after being coated with PDA and AgNPs (1A). The in vitro cytotoxicity of advanced BC–PDA–AgNPs composite towards NIH-3T3 cells displayed its biocompatibility. The existence of AgNPs further shows the excellent antibacterial activity against Gram-positive (*Staphylococcus aureus*) and Gram-negative (*E. coli*, *Pseudomonas aeruginosa*, *and Klebsiella pneumoniae*) bacteria. BC–PDA–AgNPs significantly promoted the fibroblast proliferation, granulation tissue formation, angiogenesis, and re-epithelialization, as demonstrated in histopathological examination. Gene expression studies have revealed the molecular mechanism behind the fast wound-healing rate of BC–PDAg treatment through the upregulation of IL-10, VEGF-A, VEGF-B, and bFGF and suppression of IL-1 α and IL-3 transcripts. Furthermore, TGF-β3 and SMAD-3 expression has proven the promotion of wound healing without scar formation.

**Figure 1:**
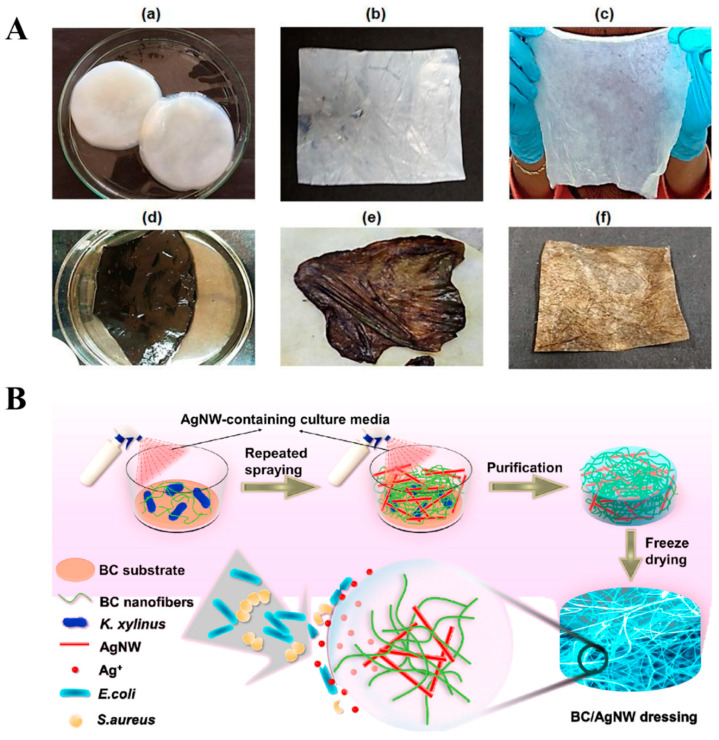
(**A**) Digital photograph images of (**a**) freshly prepared BC scaffold, (**b**) dried BC, (**c**) swollen BC, (**d**) PDA-coated BC, (**e**) AgNPs-reinforced BC–PDA, and (**f**) dried scaffold of AgNPs-reinforced BC–PDA; (**B**) schematic representation of BC–AgNWs wound dressing agent. Reprinted with permission [53,54]. Copyright 2020 Elsevier.

An alternative candidate, silver nanowires (AgNWs), has also gained much attention for use as an antimicrobial agent. One advantage of the reinforcement of AgNWs into the matrix is that they do not block skin pores to maintain air circulation. In addition to this, AgNWs do not release rapid silver ions, thereby improving the biocompatibility of the composite. By taking advantage of AgNWs, Wan et al. developed a robust and stretchable AgNWs-impregnated BC composite for wound-dressing applications [54]. As shown in Figure 1B, the process involves repeated sprays of AgNWs containing culture media during BC growth and then purification and freeze-drying. The BC–AgNWs composite could absorb wound extrudate and maintain a moist environment. The animal-testing results confirmed that a BC–AgNWs dressing with 38.4% AgNWs displayed higher expression levels of cytokeratin-10 and integrin-β4, greater proliferation of keratinocytes, and the formation of epithelial tissues, which significantly improved skin regeneration over the bare BC.

Metal oxides such as TiO_2_ and ZnO also proved their potential as antimicrobial agents by releasing of ROS. Therefore, the combination of BC composites with TiO_2_ and ZnO also displayed excellent antibacterial properties and had cellular adhesion and proliferation of fibroblast cells, thereby improving the wound-healing capability [55,56]. Similarly, Khalid et al. developed a BC–ZnO nanocomposite for the healing of burn wounds. The composite displayed bacterial killing of about 90%, 87.4%, 94.3%, and 90.9% against *Escherichia coli*, *Pseudomonas aeruginosa*, *Staphylococcus aureus*, and *Citrobacter freundii*, respectively. The composite-treated animals showed significant healing activity (66%) [57].

#### 2.2.2. Cartilage Tissue Engineering (CTE)

The regeneration of articular cartilage damage is an area of great interest due to the limited ability of cartilage to self-repair. The latest cartilage repair strategies are dependent on access to biomaterials to which chondrocytes can attach and in which they can migrate and proliferate, producing their extracellular matrix. Svensson et al. reported the production of BC secreted from Gluconacetobacter xylinus (*G. xylinus*) and its potentiality for the cell proliferation of cartilage chondrocyte [58]. BC was chemically modified with phosphorylation (PBC) and sulfation (SBC) to mimic the glucosaminoglycans of native cartilage. The results demonstrated that unmodified BC supports better chondrocyte cell proliferation (~50%) than chemically modified BC, alginate, and culture plastic. The modification of BC provided a highly compact network structure, which is unable to provide the chondrocyte cells. Therefore, BC is a suitable scaffold for CTE. 

The metabolically engineered cellulose (MBC) was developed from *G. xylinus* growth under N-acetylglucosamine (GlcNAc) conditions, which has low crystallinity and lysozyme susceptibility as compared to native BC [59]. By considering the advantage of the glucosaminoglycan-like chemistry of MBC, Yadav et al. studied the MBC scaffold for CTE. The culturing of hMSCs on MBC exhibited the suitable attachment, proliferation, and differentiation of hMSCs in MBC [60]. After 4 weeks, the cultivation of cells in MBC resulted in a spatial cell arrangement, and collagen type-II and ACAN distribution in MBC for 4 weeks cultivation of cells represents the potentiality of MBC in CTE.

**Figure 2:**
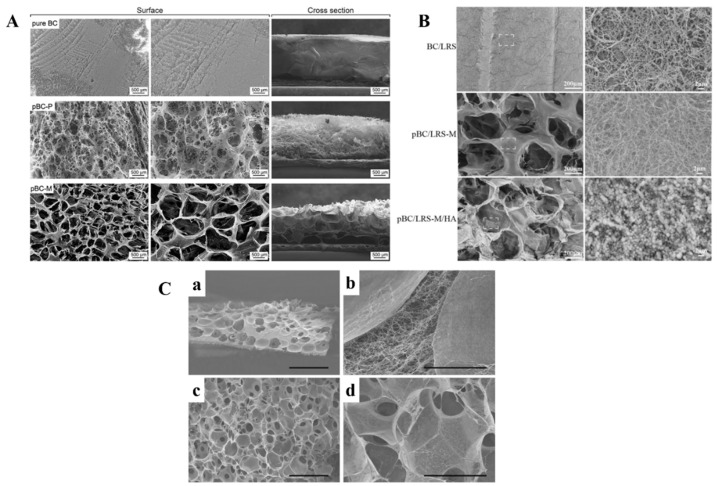
SEM images of (**A**) pure BC, pBC-P, and pBC-M, (**B**) BC–LRS, pBC–LRS-M and pBC–LRS-M–HA scaffolds; (**C**) (**a**) cross-section images of porous BC tube, (**b**) close up view of BC wall between pores, (**c**) porous structure of outer side, and (**d**) higher-resolution image of small area on the outer side of BC scaffold. Reprinted with permission [61,62,63]. Copyright 2015 Elsevier, 2019 Springer Nature, and 2010 John Wiley and Sons.

The scaffold with good porosity, pore size, and a highly interconnected network structure is important for improving cell growth and penetration. BC alone cannot provide an excellent environment for cartilage cell growth and penetration because of the pore size of the material, i.e., ~0.02–10 um. Therefore, improvement in the porosity of the scaffold is necessary for CTE. Several methods have been implemented for improving the pore size and interconnectivity of BC scaffolds. Yin et al. developed agarose particle-templated BC scaffolds with pore sizes between 300 and 500 um. In this method, monodisperse agarose microparticles (diameter range from 300 to 500 um) were layered on growing BC pellicles via upward movement of *Acetabacter xylinium* cells (*A. xylinium*) [61]. The scanning electron microscopy images represent the increased pore size after removing progen (agarose) particles by the autoclave treatment method compared to the native BC production method (Figure 2A). The BC scaffold with a pore diameter between 300 and 500 um obtained from this study could improve the chondrocyte proliferation and differentiation. Moreover, the chondrocyte cells cultured on porous BC scaffold enhanced the growth of cells for 14 days longer than native BC scaffold.

In another report, an in situ method was used to develop a porous scaffold by incorporating lotus-root starch into BC (produced from *A. xylinum*), and spherical agarose particles were used as progen particles [62]. The scaffold was further mineralized to reinforce the HAp on the surface. The scaffold pBC–LRS-M–HA has a nanoporous hierarchical structure with a pore size of 300–500 µm (Figure 2B). The composite scaffold had a highly interconnected network with porous structures, enabling efficient chondrocyte cell distribution with higher cell densities after 14 days of incubation. A highly porous BC scaffold was developed via in situ growth of *A. xylinum* cells in slightly fused particles as progen with a diameter of 150–300 um [63]. The progen was quickly removed by the extrusion method. As seen in the SEM image of BC scaffold developed from this method (Figure 2C), a highly porous structure with a highly interconnected network structure was formed. The cross-section image shows that the pore size is about 150–300 um. The resulting scaffold provided a suitable environment for articular chondrocyte cell growth from young adult patients, and neonatal articular chondrocytes were grown in porous BC scaffolds. Another benefit from this study is that the chondrocyte cells produced glycosamino glycon ECM matrix within the scaffold. Therefore, the improvement of porous structures within the BC scaffold provides suitable candidates for cartilage regeneration.

Akaraonye et al. developed a 3D-composite scaffold composed of poly(3-hydroxybutyrate) (P(3HB)) and micro-fibrillated bacterial cellulose (BC) using progen (sucrose) leaching [64]. Using this method, high dispersion and strong adhesion between P(3HB) and BC was achieved to improve properties such as load bearing, surface area, pore diameter, and morphological resemblance to the native ECM. The availability of large pore-sizes (60–83 um) in P(3HB)–BC 3D-composite scaffold provided suitable cell attachment and proliferation, with deep infiltration and migration of mouse chondrogenic ATDC5 cells. 

A macroporous scaffold was developed using a simple freeze-drying technique for BC suspension followed by crosslinking using 1-ethyl-3-(3-dimethylaminopropyl)carbodiimide hydrochloride/N-hydroxysuccinimide (EDC/NHS) [65]. The scaffold exhibited excellent compression and shape-recovery properties. The average pore size of the scaffold is about 200 um, which can enable cell growth compared to BC scaffold alone. The in vivo result showed that the neocartilage tissue with native cartilage appearance and abundant cartilage-specific extracellular matrix deposition is successfully regenerated in nude mice.

Gu et al. developed methacrylate gelatin (mGT)–BC composite hydrogels using the photo polymerization technique [66]. The porosity can be tuned by increasing the BC content in the prepolymerized solutions. The composite hydrogel had a highly interconnected network with the pore size decreased from 200 um to 10 um by increasing the BC content (0, 1, 2, 4, and 8 % BC). The hydrogel is toughened, and the compressive moduli range from 112.9 ± 15.4 to 811.7 ± 23.4 kPa by increasing the BC content, which was close to human articular cartilage [67]. The chondrocyte cells encapsulated within the mGT–BC composite hydrogel maintained their chondrocyte phenotype for 7 days of culture incubation.

So far, various microcarriers have been developed using natural polymers such as GT, COL, SA, and chitosan (CS) for cartilage regeneration. The major disadvantage of using microcarriers in CTE is the limitation in the diffusion of nutrients and oxygen. Moreover, natural microcarriers have limitations in vivo, such as irregular specific surfaces, low mechanical strength, and fast degradation rate [68]. BC is advantageous for CTE because of its mechanical properties and structural similarities to the native ECM. Initially, aldehyde functionalized BC was synthesized using the oxidation method and then modified with hydroxylysine (DHYL), which is a collagen type-II component; hyaluronic acid (HA); and CS via Schiff’s base reaction [69]. In this approach, microcarriers provided good porosity and pore size, as well as the mechanical property of a uniform pore diameter. Cartilage micro-tissue was developed by culturing bone-marrow-derived mesenchymal stem cells (BMSCs) with BC fibrous micro-carriers using a rotary cell culture approach for 21 days. In mouse models, the developed microtissues were implanted into a critical-size knee articular defect and showed improved cartilage tissue regeneration. 

Horberrt et al. developed a new approach for 3D-laser perforation of BC implants (derived from *G. xylinus*) seeded with chondrocyte cells and free in a cartilage punch model [70]. The movement and ingrowth of chondrocyte cells in BC implants provided long-term performance in the regeneration of cartilage. The cartilage regeneration started by changing the appropriate cell seeding composition and its differentiation process. 

**Figure 3:**
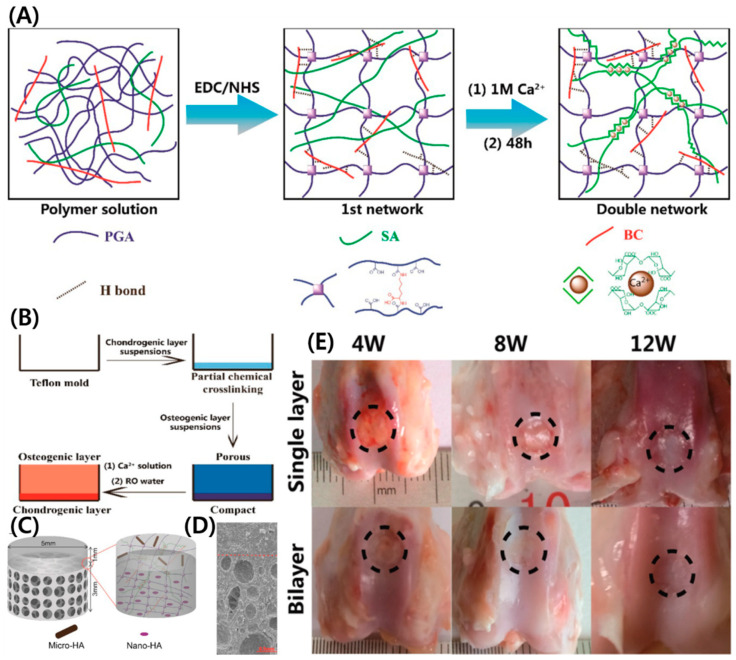
(**A**) Schematic representation of the formation of BC–DN hydrogel, (**B**) bilayered hydrogel, (**C**) structure illustration of bilayered hydrogel, (**D**) SEM image of bilayered BC hydrogel, (**E**) digital images of knees repaired at different timpoints. Reprinted with permission [71]. Copyright 2018 American Chemical Society.

A double-network polymeric architecture has unique advantages for TE applications as they provide good mechanical compliance, similar to native ECM. Zhu et al. developed DN hydrogels composed of BC with lysine crosslinked with poly(gamma-glutamic acid) (coupling reaction) and ionically crosslinked SA (Ca^+2^) to improve the mechanical property [71]. The DN hydrogel system consists of a bilayer with a nonporous top layer and porous bottom layer, structurally similar to native osteochondral tissue. To improve the osteochondral tissue regeneration, the DN hydrogel-reinforced HA microparticles on the top layer and HAp nanocrystals on the bottom layer (Figure 3A–D). Furthermore, the prepared DN hydrogel showed good repairability for the osteochondral defect model of rabbits. As shown in Figure 3E, the implanted single-layer and bilayer hydrogel showed a granulation tissue for the single-layer group, whereas fibrous tissue was observed for the bilayer group at 4 weeks. At 8 weeks, surrounding tissue could be seen around the implant for a single layer, while it almost disappeared for the bilayer group. At 12 weeks, surrounding tissue disappeared for the single-layer group, while newly formed chondral tissue in the bilayer group was closer to native tissue. From this study, a simple approach provided biomimetic bilayer hydrogel with a good effect and significant advantages in osteochondral repair.

In another study, Kumbhar et al. developed a bilayer composite composed of BC–HAp and BC modified with glycosaminoglycons (GAG) [72]. It was shown that the designed scaffold could support cell adhesion and proliferation in vitro. The in vivo tests were also performed by implanting bilayer composite in the osteochondral defect site of rat knees. The bilayer scaffold accelerated the subchondral bone as well as articular cartilage. The degradation of intervertebral discs (IVD), a typical hierarchical structured tissue, causes severe neck and back pain. The current methods cannot fully reconstitute the unique structure and function of native IVD. A new approach has been developed by Yang et al., who proposed to prepare 3D structures mimicking intervertebral discs [73]. In this method, *A. xylinum* was cultivated in a micropatterned polydimethylsiloxane template, then BC membranes were rolled to form the fibrous structure of an intervertebral disk and filled with a collagen. The in vitro results demonstrated the biocompatibility of BC membranes. The in vivo test on rats further demonstrated that the implant has good shape maintenance, hydration, tissue integration, mechanical support, and flexibility for the segment to move.

#### 2.2.3. Bone Tissue Engineering

It is well known that bone is mainly composed of collagen (COL) and hydroxyapatite (HA) nanocomposites. To date, many researchers have developed suitable composite materials to mimic naturalistic bone environments for bone regeneration. Therefore, the combination of HA with BC scaffold could provide a biomimetic environment and is expected to offer a novel bone implant. In addition, growth factors also play an important role in the bone maturation of preosteoblast cells or mesenchymal stem cells. So far, many research groups have been actively developing various BC scaffolds with the combination of other natural/synthetic polymers, nanofillers, and growth factors to meet the naturalistic environment for bone regeneration. 

The BC scaffold quickly provides structural support to the mesenchymal stem cells (MSCs) for proliferation and potential differentiation into osteocytes and chondrocytes using differentiation media [74]. The mineralized macroporous BC scaffold coated with calcium phosphate also provided proliferation and differentiation of MSCs towards the osteoblastic phenotype [75]. To improve the osteogenic potential of bone cells, the BC scaffold was further modified or loaded with osteogenic growth factors. Osteogenic growth peptide (OGP) is a native molecule that is identical to C-terminal sequences of histone H4. The OGP can easily regulate the osteogenesis structure of osteoprogenitor cells [76]. The BC-membrane-functionalized OGP was synthesized for BTE. The in vitro culturing of CHO-K1 cells cultured on OGP–BC membranes resulted in enhanced osteoblastic phenotype characteristics [77]. The BC scaffold from *Acetobacter xylinum X-2* loaded with BMP-2 had a suitable osteogenesis property for BTE. The data showed that BC–BMP-2 scaffold induced the differentiation of mouse fibroblast-like C2C12 cells into osteoblasts by in vitro conditions. The BMP-2-coated BC scaffold showed alkaline phosphate activity. The implantation of BMP-2-coated BC scaffold to a rat subcutaneous model (Male Sprague–Dawley (SD) rats weighing 180–200 g) showed more bone formation and higher calcium levels within 4 weeks [78]. In another study, Dubey et al. developed a 3D scaffold composed of microporous/nanofibrous bacterial cellulose from *Komagataeibacter europaeus SGP37* loaded with BMP-2 protein as a therapeutic agent [79]. Low-dose BMP-2 primed murine mesenchymal stem cells (C3H10T1/2)-seeded scaffold with preconditioned MBP-2 (50 ng/mL) resulted in osteodifferentiation of cells within 3 weeks. The results showed an early onset and significantly enhanced bone matrix secretion and maturation in the scaffolds seeded with BMP-2-primed cells compared to the unprimed ones. These findings suggest that with the aid of ‘osteoinduction’ from low-dose BMP-2 priming of stem cells and ‘osteoconduction’ from nano-macro/micro-topography of NBC scaffolds, a cost-effective bone tissue engineering strategy can be designed for quick and excellent in vivo osseointegration. 

For BTE, the scaffold fabrication from composite materials is essential for improving the physicochemical properties to meet the native tissue environment. Hydroxyapatite (HA) bioactivated BC scaffolds were developed by Tazi et al. to promote osteoblast growth and bone nodule formation. In that study, the combination of BC and HA was shown to significantly improve osteoblast adhesion and growth [80]. Moreover, alkaline phosphate assay revealed the BC–HA could enhance the ALP levels (5.3 mM) as compared to BC alone (2.5 mM).

The combination of polycaprolactone (PCL), gelatin (GEL), BC, and HA as composite materials were used for a 3D-printed scaffold for bone tissue engineering [81]. Three-dimensional printing is a promising method for bone tissue engineering. In this study, the mean pore size of scaffold increased from 301.67 + 46.3 to 304.77 + 26.9 um by the addition of BC in PCL–GEL scaffold, which is close to the optimal pore sizes for osteoconduction. The 3D-printed PCL–GEL–BC–HA scaffold provided 80% porosity, enabling improved cell functions such as proliferation and attachment of human osteoblast cells. 

In another report, Gutiérrez-Hernández et al. developed a composite scaffold composed of BC and functionalized multi-walled carbon nanotubes (F-MWCT) [82]. This work aims to improve the mechanical property of the BC composite scaffold by the reinforcement of F-MWCT. Reinforcement improved the osteoblast cell viability, proliferation, and adhesion. Huang et al. developed a porous BC scaffold modified with gelatin (GT) via a crosslinking method using procyanidins (PA) as crosslinker. Furthermore, HAp was also reinforced with a modified BC scaffold [83]. The mechanical testing in Young’s modulus, maximum load, and compressive strength were significantly more improved for BC–GT–PA–HA composite scaffold than BC alone, BC–GT, or BC–PA–GT. The in vitro and in vivo results confirmed the bone-formation ability either in nude mice or rabbits. Therefore, an appropriate crosslinking method can positively influence properties such as the physicochemical and biological properties. 

In general, the physical complex method formed between BC and COL via non-covalent interactions resulted in poor stability [84]. The crosslinker glutaraldehyde (GA) stabilized the composite via chemical bonds formed between GA and components [85]. However, the biological toxicity of GA limits its application in TE. The BC modified with glycine combined with COL formed an esterification bond, thereby improving the composite stability. The problems that arose from the use of too many reaction steps were difficult to control. Zhang et al. used a simple Malaprade and Schiff’s-base reactions for the formation of stable BC–I COL composite loaded with BMP-2 for bone TE [86]. The combination of components and reactions formed stable 3D-porous microspheres of BC–COL–BMP-2. The microspheres had rough surfaces with porous structures and a particle size of about 198.5 nm. The microspheres showed good biocompatibility and promoted the adhesion, proliferation, and osteogenic differentiation of mice MC3T3-E1 cells. In another report, Saska et al. developed a composite based on BC, COL, HAP, and osteogenic growth factor (OGP) for BTE. In this report, the nanocomposite stabilized by using a carbodiimide coupling agent [87]. The nanocomposite route was effective for bone-like apatite formation without affecting the osteoblast cell viability. Additionally, the growth factor enhanced the growth of osteoblast and its maturation for effective bone regeneration. 

Bacterial cellulose–hydroxyapatite (BC–HAp) composite had good bioaffinity, but its poor mechanical strength limited its widespread applications in bone tissue engineering (BTE). The development of a double-network (DN) composite provides better mechanical properties for BTE. For this, Ran et al. developed an organic–inorganic multicomponent composite using BC, gelatin, and hydroxyapatite combination [88]. The wet BC–GT–HA composite showed a higher elastic modulus (0.27 MPa) than BC–GT (0.12 MPa). The dry BC–GT–HA composite had higher a mechanical strength and stiffness (177 MPa and 12.95 MPa, respectively) than the BC–HA composite (48 MPa and 7.81 MPa). The improved mechanical properties are advantageous for BTE. Moreover, the GT molecules that existed in BC could be completely dissolved in PBS solution within 20 days. The in vitro evaluation of rBMSCs cultured on BC–GT–HA composite had better adhesion, proliferation, and differentiation. Moreover, the BC–GT–HA composite presented higher ALP activities as compared to other compositions (BC, BC–HA, and BC–GT) for applicability in BTE.

To promote better bioactivity towards the BC, Coelho et al. developed a BC composite incorporated with HA followed by loading of antibody-BMP-2 [89]. The composite shows excellent biocompatibility towards MC3T3-E1 cells. The release of antibody-BMP-2 from composite BC membrane resulted in a 70% release in 7 days and 90% in 14 days. The composite-treated bone cells showed enhanced bone mineralization and alkane phosphate activity (ALP). Therefore, in vivo study is necessary for the verification action of this combination for future studies.

Osteopontin (OPN) is an extracellular glycosylated phosphoprotein that is abundantly found in the mineral–tissue interface of bone with molecular weight ranges from 41–75 kDa, owing to the presence of two essential amino acid sequences (RGD and SVVYGLR) that mainly interact with various αv integrin receptors and play significant roles in cell attachment and bone formation [90]. The plant-derived rhOPN (p-rhOPN) resembles the rhOPN derived from mammalian cells. By considering the OPN role in bone formation, p-rhOPN conjugate on PAA-grafted BC was synthesized via amide bond formation. The immobilized p-rhOPN promotes the oseogenic differentiation of PDLSCs cells as compared to rhOPN-immobilized PAA-grafted BC. Overall, p-rhOPN-immobilized PAA-grafted BC has greater potential in BTE, although this requires further confirmation. Moreover, there are still studies required to confirm the bone-regeneration mechanism using p-rhOPN by in vivo [91]. 

**Figure 4:**
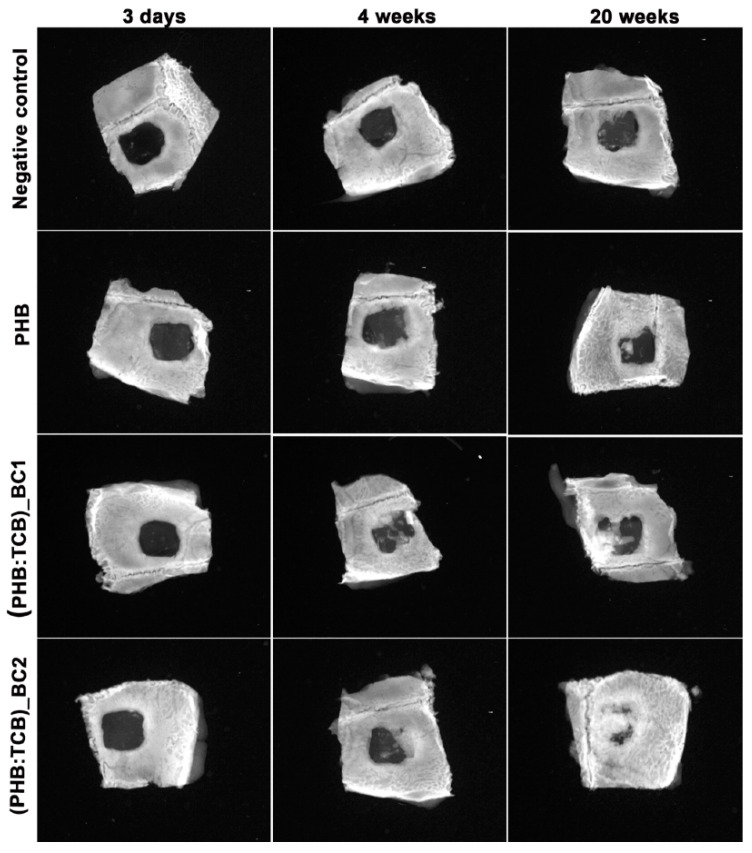
Radiographs of in vivo bone samples treated with scaffolds at different days (3 days, 4 weeks, and 20 weeks). Reprinted with permission [92].

Codreanu et al. developed BC-modified polyhydroxyalkanoates (PHB–BC) scaffolds for osteogenic potential in critical-size mouse calvaria defects [92]. The scaffolds were prepared using salt leaching by varying the PHB, BC, and tributyl citrate (TBC) using NaCl as salt. The scaffolds can support viability and proliferation for 3T3-L1 preadipocytes. BC reinforced that PHB had enhanced osteoblast differentiation in vitro in the first 4 weeks post-implantation. As shown in Figure 4, the radio-opacity in the mid-region of the defect was low, representing less mineralization at this time interval. The ossification is advanced at 20 weeks due to the new bone deposition and growth and more advanced for PHB: TBC–BC2 (PHB-76 wt%, TBC-19 wt%, and BC-2 wt%) as compared to other formulations.

#### 2.2.4. Dental Implants

The integration of dental implants into the surrounding tissue is a major problem in dental implants. In addition to this, complete osseointegration between implant and bone is necessary for bone regeneration. Owing to the high absorptive capacity, good volume retention, and good mechanical strength of BC, it could be a choice for commercial interests in dental applications. A few studies have evidenced the BC as dental canal treatment or oral fields, as it provides absorption, tensile strength expansion, and biological features compared to other paper point materials. The BC maintains excellent absorption and expansion capacity and maintains good mechanical properties under wet conditions [93]. 

An et al. developed BC membranes for guided bone regeneration. An electron beam irradiation was applied to break glucose bonds of BC [94]. The EI–BC was assessed in vitro cytocompatibility of NIH3T3 cells. The in vivo biodegradability and bone regeneration results of EI–BC membrane in rat calvarial models for 4 and 8 weeks resulted in their effective interactions with cells and promoted bone regeneration. Similarly, Pigossi et al. developed a potential osteogenic grown peptide (OGP) integrated BC–HAp composite for bone regeneration of critical-size calvarial defects in mice [95]. The integration of OGP enhanced the gene expression of bone biomarkers such as Tnfrsf11b and Alpl, Spp at 60 and 90 days, which leads to the potentiality of composite for bone regeneration in critical-sized calvarial defects in mice. 

The placing of the dental implant in the posterior maxillary region has difficulties in the height of the alveolar process. Koike et al. developed BMP-2-loaded BC to allow effective alveolar bone augmentation; BC maintained the graft space and released BMP-2 in a sustained manner, promoting optimal bone formation [96]. The combination of BC–BMP-2 could enhance bone regeneration and be useful for clinical pre-dental implant-bone augmentation in the maxillary sinus.

On the other hand, surgical wounds of the oral mucosa are important issues in dentistry. Small defects usually heal primarily on closure, but split or full-thickness grafts can be used for moderate ones. For defects involving most of the buccal mucosa, there is a need for a second surgical area. For this, Chiaoprakobji et al. prepared a novel three-dimensional composite composed of BC–SA (BCA). In this study, HaCat cells (keratinocyte cell line) were seeded on the BC scaffold and BCA scaffold [97]. The results conclude that the BCA scaffold has good potential use in the oral cavity to cover surgical wounds. Similarly, Jinga et al. used cellulose whiskers with commercial mineral trioxide aggregate as a reinforcement material [98]. The study showed that BC accelerated the hardening process of the mineral trioxide aggregate cement and decreased the relative quantity of calcium hydroxide crystals.

#### 2.2.5. Artificial Blood Vessels and Vascular Grafts

Vascular grafts are used to bypass damaged or diseased blood vessels. So far, Dacron, ePTFE, and polyurethane are the most commonly used materials for artificial blood vessels. Recent studies have shown that BC is a promising material for preparing artificial blood vessels. BC has been studied for use as an off-the-shelf graft. BC is a polysaccharide produced by *Gluconacetobacter xylinus* and *Acetobacter xylinum* bacteria with interesting properties for arterial grafting and vascular tissue engineering, including high-burst pressure, high-water content, high crystallinity, and an ultrafine highly pure fibrous structure similar to that of collagen [99]. 

In vascular replacement, the mismatch is a major problem for the development of intimal hyperplasia. Appropriate mechanical property is required for BC production to support cardiovascular grafts. Zahedmanesh et al. developed BC tubes to know the potentiality of vascular graft models [100]. The BC tubes showed a good compliance response, more similar to the human saphenous vein (4.27X10-2% per mmHg over the 30–120 mmHg) than that of other commercial Dacron and ePTFE saphenous vein products. The culturing of endothelial cells and bovine smooth muscle cells on BC showed good biocompatibility and adherence. 

**Figure 5:**
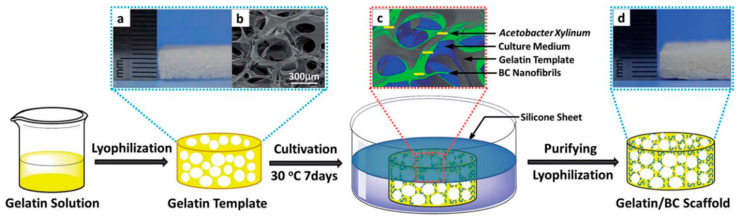
Schematic representation of GEL-BC by template synthesis method: (**a**) digital photograph image of GEL microporous template; (**b**) SEM image; (**c**) BC nanofibrils formed by biosynthesis method using G. Xylinus in GEL microporous template; (**d**) digital photograph image of GEL-BC scaffold. Reprinted with permission [102]. Copyright 2016 Royal Society of Chemistry.

BC nanofiber is a potential material for the prevention of blood clots. Therefore, it is considerable for artificial blood vessels or vascular tissue engineering. The surface modification of heparin with other materials is important for vascular tissue engineering because of its anticoagulant properties. In addition to this, it can also easily bind with angiogenic growth factors such as vascular endothelial growth factor (VEGF) and fibroblast growth factor 2 (FGF-2) to promote the growth of blood vessels. Thus, BC modified with heparin can be utilized for vascular tissue engineering [101]. In general, native BC nanofibrous scaffold use for tissue regeneration has limitations due to the formation of nanopores that inhibit the cell infiltration and vascularization in 3D scaffold. To overcome this problem, Li et al. developed a 3D scaffold with the combination of gelatin and BC to create micropores by the template biosynthesis method [102]. As from Figure 5a–d, a microporous GEL template with highly interconnected micropores (171 ± 71 um) was fabricated by lyophilization followed by *Gluconacetobacter xylinus strain* cultivation at 30 °C for 7 days. During the cultivation, BC nanofibers (25.2 ± 7.0) were formed along with a microporous GEL template scaffold. The structure and mechanical properties of the developed 3D microporous nanofibrous scaffold (GEL–BC) are similar to native ECM, enabling the growth of adipose-derived stem cells (ADSCs). The preliminary in vitro and in vivo biocompatibility results proved the improving cellular infiltration and vascularization compared to BC and GEL scaffold.

Although BC has excellent biocompatibility and mechanical strength, the large tissue construct for in vivo remains a challenge due to insufficient vascularization. For this, Sämfors et al. developed a BC scaffold with a vascular lumen structure [103]. Three steps have been created for the potential performance of the BC scaffold. In the first step, BC scaffolds consist of channeled structures that could create the growth of human umbilical vein endothelial cells. The second step involved constructing a vascular tree using a 3D printer, which consists of a nanoporous BC scaffold lined with endothelial cells. In the final step, an interconnected macroporous lumen structure was created. The method described in this study produce larger constructs containing channeled structures for the kidneys. 

Coronary artery bypass grafting is routinely used around the world. For this, autologous internal mammary artery and saphenous vein grafts have been used. The use of a small saphenous vein is not suitable because of varicosities. Therefore, it is important to create small-diameter vascular grafts. By taking advantage of BC production with the different shapes using molds, it is possible to develop small-diameter vascular grafts to meet mechanical and biological properties to match native tissues. Schumann et al. developed a small-caliber vascular graft with a diameter of 1.0–3.7 mm, length of 5.0–10.0 mm, and wall-thickness of 0.7 mm for small arterial substitutes [104]. The in vivo experiment of the developed BC graft was used to perform end-to-end anastomosis for the carotid artery in the white rat. From this experiment, there was no bleeding and no arteriovenous formation from grafted material. Moreover, there were no signs of inflammation around the graft that could be observed after 3 months. The method of fabrication of BC grafts is an attractive approach for cardiovascular surgery. 

In another study, Scherner et al. developed a small-diameter blood vessel of tubular BC hydrogels with fibrous network structures using Gluconacetobacter strain and matrix reservoir technology [105]. The BC tube dimensions (100 mm length and 4.0–5.0 mm inner diameter) were implanted to replace the carotid arteries for functional in vivo performance such as neoformation of a vascular wall and inflammatory potential. The developed BC graft had a bursting strength of ~800 mm Hg and a suture retention strength of 4–5 N, sufficient to implant graft to the high-pressure bloodstream. Thus, the BC graft provides a scaffold for cell growth and support. 

The nonelastic BC membranes produce tube-like structures supported by strips. For implantation, the strips could be removed; however, they sometimes change their shape after removing the strips. A new method has been described for the preparation of small-diameter BC tubes, in which cells are loaded on the membrane and then rolled. This is a phenomenon of interest to mimic the layers of the vessel. The in vivo implantation suggests that thrombi formation and immune cells were absent between the layers of the BC tube after 21 days. The results confirmed the potentiality of small-diameter vascular grafts using this approach. However, further experiments would require performing cell-laden grafts in the future [106].

Leitao et al. developed a simple, cost-effective method for producing small-caliber BC graft vascular prosthesis using the capillary drying, shaping, and freeze-drying method [107]. The technique allows the formation of BC tubes with a 2 mm wall thickness. The molecular interactions (H bonds) could form the spatial orientation of BC fibers. The in vivo test was further performed by the implantation of developed BC grafts. The preliminary in vivo assay was performed with the implantation of BC grafts in a homolateral-femoral artery bypass on the left hind limb of female domestic pigs. The results demonstrated no signs of occlusion.

Tang et al. developed BC tubes composed of poly(vinyl alcohol) (PVA) and BC by a thermally induced phase-separation method to improve the properties for artificial blood vessels [108]. The PVA impregnated into BC tubes shows significant improvement in mechanical and water permeability properties. BNC tubes significantly improved the properties of BNC, especially the mechanical properties and water permeability. However, biological studies would be required to assess the potentiality of BC tubes for artery transplantation. 

**Figure 6:**
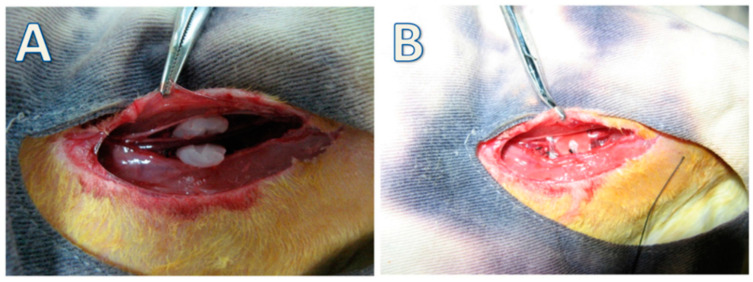
(**A**) The implanted BC graft in rabbit femoral artery and (**B**) their suture of the trauma. Reprinted with permission [109]. Copyright 2015 Elsevier.

PDMS has high oxygen-permeability that could be considered a template for the production of BC tubes with dimensions of 100 mm, an outer diameter of 4 or 6 mm, and a thickness of 1 mm. Zang et al. developed tube-like artificial blood vessels from BC using Gluconacetobacter xylinum with a PDMS tube as the template [109]. The Young’s moduli of 4 mm and 6 mm BC tubes were 3.94 + 0.12 MPa and 3.30 + 0.19 MPa, respectively. The mechanical property is comparable to the porcine carotid artery (1.04 MPa). The BC tube has no toxic effect on vessel-related cells cultured on their surface (endothelial, smooth muscle, and fibroblast cells). Finally, BC tubes were implanted in New Zealand rabbits weighing between 2.5 and 3 kg. The target artery was wrapped with the BC tubes by interrupted suture with 7/0 prolene monofilament (Figure 6). The results confirmed the in vivo study proved the BC tubes exhibit complete endothelialization. Therefore, BC tubes appear to be appropriate for applying vessel transplantation and reconstructive surgery.

#### 2.2.6. Urethral Implants

The urethra is a hollow organ that structurally consists of two layers. A stratified epithelium upper layer provides a barrier for the urethra, and the organized ticker subcutaneous layer contains fibroblasts and muscle cells that provide flexibility and strength [110]. Several diseases damage the urethral function. A TE approach could be promising for urethral regeneration. In UTE, decellularized matrixes and synthetic polymer materials have been applied for urethral reconstruction. However, these scaffolds could not provide better barrier function urine. By considering the dense network structure of BC and its mechanical property, similarity with native tissue could be considered for UTE. A few efforts have been studied on BC for urethral regeneration. Lima et al. reported the application of a BC membrane as a protective urethral barrier in female Wistar rat animal models [111]. The results showed that the BC membrane was well integrated into the urethral wall, promoting tissue remodeling. In another report, Maia et al. also used a BC membrane as a urethral reinforcement for ureterovesical anastomosis in rabbit models [112]. The results conclude that there was a decrease in wall thickness after 14 weeks of implantation.

A major challenge in UTE is mimicking the scaffold architecture to the native complex structure of the urethra. For this, a bilayer scaffold comprises the smooth upper layer and porous sublayer, which could mimic the native tissue-like structure. Lv et al. developed a bilayer scaffold composed of potato starch (PS) and BC [113]. In this approach, PS was initially gelatinized and then inoculated using *G. xylinus* to form a dense top layer. The scaffold easily allows the muscle cell infiltration in the loose layer and enhances wound healing in vivo in dog urethral defect models. Organized muscle bundles and the epithelial layer were observed in animals treated with BC–PS scaffold. The same group developed a bilayer scaffold composed of SF microporous scaffold and BC nanoporous dense network [114]. In this study, SF microporous scaffold was prepared by freeze-drying technique followed by inoculation in *G. xylinus*. A dense BC network was formed on the upper layer of the SF scaffold. The scaffold architecture BC layer prevents the erosion of toxic substances in urine and is capable of promoting the growth of epithelial cells. The SF layer is beneficial for the migration of cells. The study provided preclinical evidence for BC scaffold architecture for the reconstruction of urethral defects. However, more studies are required to prove the feasibility of this concept in UTE.

#### 2.2.7. Artificial Cornea and Retina

Corneal disease is the second leading cause of blindness. A corneal transplant is an optimal treatment for patients which corneal blindness. Although corneal transplantation is well known and successful clinically, several drawbacks include shortage of corneal tissue, graft failure, and immune rejection. TE is an alternative for tissue regeneration. BC has recently been shown to be promising as a potential scaffold for cornea stromal cell growth. So far, a few reports have been attempted for corneal regeneration. Sepúlveda et al. developed BC and BC–PCL membranes that could be successfully implanted in rabbit cornea [115]. BC and BC–PCL implants were stable in corneal tissue and protected ocular surfaces for 45 days of follow-up time. The results demonstrated that a moderate inflammatory response was observed due to incomplete epithelialization over the implanted membranes and disorganized collagen fibers.

Zhang et al. studied the biocompatibility evolution of BC scaffold for corneal stroma TE. The biocompatibility of the BC scaffold was evaluated using rabbit corneal epithelial cells and stromal cells [116]. The BC membrane showed a nanofibrous structure with high transmittance properties. Figure shows the BC grafting in corneal graft. The insertion of BC is transparent, and no infiltration in the cornea was observed under a slit lamp. After 7 and 30 days post-surgery, both the cornea and BC appeared transparent. After 90 days post-surgery, they remained transparent. Therefore, BC had good biocompatibility for corneal stroma TE.

Anton-Sales et al. reported an initial study of the ability of BC for a corneal bandage. BC exhibits more extended stability under in vitro and ex vivo conditions than amniotic membrane [117]. Additionally, it could offer good conformability to the shape of the eye globe and easy manipulation in medical settings.

The retinal pigment epithelium (RPE) is a highly polarized monolayer of polygonal-shaped and pigmented epithelial cells with apical microvilli and basolateral infoldings [118]. With age, the RPE may be affected, compromising retinal integrity and ultimately leading to retinal degenerative diseases such as age-related macular degeneration (AMD)—the most common cause of blindness worldwide [119]. A promising therapeutic approach for AMD involves replacing diseased RPE with healthy stem-cell-derived RPE-like cells, transplanted as an integer epithelial sheet on a carrier substrate [120]. Since the Bruch’s membrane (BM) is also affected in AMD, the success of RPE transplants requires the replacement of this tissue, as these RPE cells are transplanted in a BM prosthetic substrate. The requirements of an ideal BM prosthetic substrate for RPE transplantation are very complex; therefore, the development of viable substrates has not yet been successful. BC may be a promising material for TE. The surface medication of BC via acetylation (AcBC) showed good cell attachment and proliferation [121]. In another report, Goncalves et al. developed AcBC-coated with urinary bladder [122]. The developed substrate exhibited low swelling properties, improved mechanical strength, and nopyrogenicity, which enable the adhesion and proliferation of RPE cells forming cell monolayer as similar to natural RPE cells. Moreover, RPE cells expressed metabolic RPE65 and cytoskeletal (ZO-1) essential proteins.

#### 2.2.8. Nerve Implants

Biocompatible neural interfaces hold great promise for treating neurological disorders and enhancing human beings’ mental and physical abilities. Most of the currently available neural interfaces are made from rigid, dense inorganic materials that cause tissue damage. Recently, natural cellulose has been considered an attractive candidate for neural implant materials due to its high flexibility, biocompatibility, and low cost. As mentioned above, BC can be considered an excellent candidate for tissue engineering. The use of BC in neural implants is advantageous compared to other synthetic polymers because of its superior compliance with a Young’s modulus similar to native neural tissues, extreme flexibility, improved conformational contact with curvilinear surfaces of neural tissue, high biocompatibility, and long-term subcutaneous implantation ability to record neural signals [123]. Yang et al. developed microchannel microarrays of Au deposited on BC (Au–BC). The results demonstrated the Young’s modulus of BC (120 kPA) in between brain tissue (2.7–3.1 kPa) and peripheral nerve tissue (580–120 kPa). The bending stiffness of Au–BC is similar to the Au-polyimide electrodes. The Au–BC holds great promise for neural interfaces, as evident from the recording brain electric activity in vivo [124].

The tissue engineering approach is a promising technology for the regeneration of the injured nervous system to replace the limitations of autograft. Petile et al. demonstrated that the BC functionalized recombinant proteins improved the mesenchymal stem cell adhesion, supported cell viability, and expressed the neurotrophin, promoting neuronal regeneration. Future studies (in vivo) require confirming its potentiality in nerve regeneration [125]. Kim et al. developed a graphene-oxide–BC composite by the controlled dispersion of GO in the culture media of BC, which can induce the crystalline cellulose nanofibrils. The GO–BC scaffold supports the neuronal cells similar to the in vivo environment of brain tissue [126].

In peripheral nerve repair, the scaffold requires bioabsorbability property. BC cannot degrade in the human body due to the lack of chemical or enzymatic processes. For this, Hou et al. developed a BC scaffold by applying different oxidation degrees using sodium periodate to improve biodegradation. The oxidized BC scaffold provides suitable physicochemical properties such as high porosity, interconnected pores, mechanical properties, and enhanced biodegradation. The scaffolds are biocompatible and hemocompatible and have potentiality in peripheral nerve tissue engineering [127].

Epidural fibrosis and adhesion are among the major causes of failed back-surgery syndromes. Biomaterials have been applied as barriers to these problems. Wang et al. developed a BC membrane as a barrier composed of exosomes from human umbilical cord mesenchymal stem cells for epidural fibrosis and peridural adhesions. The BC membrane could offer a 3D network structure and suitable mechanical properties. The in vivo study confirmed its biocompatibility and inhibited epidural fibrosis and peridural adhesions [128]. Jing et al. developed electrospun BC nanofibers using BC suspension in ionic solvents. The electrospun fibrous mat showed good biocompatibility with mouse fibroblasts. The in vivo performance of electrospun BC in rabbits confirmed more collagen fiber formation with lower early inflammatory reaction. The results demonstrated that electrospun BC is a suitable candidate for dura mater repair [129]. Deng et al. studied the combination of BC and O-carboxymethyl chitin bilayer composite scaffold for dural repair. The scaffold was crosslinked with glutaraldehyde or citric acid. The scaffold exhibited highly porous (pore size ~90–200 um) with good swelling properties and an excellent tensile strength. The scaffold is noncytotoxic on NIH3T3 cells. The in vivo study in mice indicated a mild inflammation reaction. Future clinical experiments require possible dura repair [130]. In another study, in vivo implantation of BC loaded with growth factors (epidermal and fibroblast) was studied for stroke treatment and brain and spinal cord injuries. The growth factors had an extended release from BC over 10 days. An in vitro study of neural stem cells cultured on a BC scaffold promotes their proliferation and differentiation by release growth factors from the BC scaffold. The in vivo study on mice indicated no inflammation reactions, which represent the duraplasty of BC scaffold for the treatment of spinal cord injuries [131].

#### 2.2.9. Delivery of Drug and Bioactive Agents

Over the past decade, various natural biopolymer-based hydrogels have been actively investigated for drug delivery applications [132]. Recently, the BC-based hydrogel scaffolds have been used in drug delivery applications because of their potentiality in terms of high reactive surface, high porosity, and fine network structure [37]. The BC can easily tune its responsiveness for drug release by modifying, blending, and by nanoparticle impregnation. 

Treesuppharat et al. developed BC–GT hydrogel crosslinked with glutaraldehyde (GA). The hydrogel presented high thermal stability, as well as mechanical and chemical resistance behavior. The hydrogels are swellable (400–600%) with controlled-release characteristic properties for methylene blue (MB) as a model drug [133]. 

Arikibe et al. developed BC–CS semi-interpenetrating (semi-IPN) hydrogels crosslinked with genipin [134]. The hydrogels are sensitive to pH stimuli. The swelling of hydrogels increased with an increase in the concentration of CS in hydrogels at low pH and increase in BC ratio at high pH. The release kinetics of quetiapine fumarate (QF; an antipsychotic drug used to treat schizophrenia) from BC–CS hydrogel better fitted with a Higuchi model for pH conditions. Moreover, it could follow non-Fickian and Super Case II transport mechanism. 

Graphene oxide (GO) possesses a high surface area, which can prevent premature drug release. By taking advantage of this, Luo et al. developed BC–GO ibuprofen (IBU) was loaded onto the BC–GO nanocomposites [135]. The release of IBU from BC–GO followed a non-Fickian diffusion mechanism and better fitted with Korsmeyere Peppas model. Therefore, IBU@BC–GO holds great promise in controlled release drug delivery systems.

Pandey et al. developed biocompatible BC–acrylamide (AAm) hydrogel as a pH-sensitive smart carrier for oral drug delivery of theophylline as a drug [136]. The BC crystallinity and gel fractions were decreased with increasing the concentration of NaOH in prepolymerized solutions, while the optical transparency, porosity, and pH-sensitive properties were improved. In vitro drug release confirmed the pH-sensitive property of hydrogel with higher drug release at pH 7.4 than pH 5.5, suggesting the oral drug release characteristics. The hydrogel is noncytotoxic and hemocompatible. The in vivo toxicity test on ICR mice indicates that the hydrogels are nontoxic up to 2000 mg/kg. Therefore, the pH-sensitive hydrogel makes it safer for oral drug-delivery applications. 

pH-sensitive hydrogels with mucoadhesive properties are potential candidates for oral delivery of protein. For this, Ahmad et al. developed a BC-grafted poly(acrylic acid) (BC-g-PAAc) hydrogel by electron-beam irradiation method without using any cross-linking agent [137]. Bovine serum albumin (BSA) as a model protein drug was encapsulated into hydrogels. The in vitro release study indicates the less than 10% cumulative release of BSA was observed in simulated gastric fluid (SGF). Therefore, the hydrogel is protected from an acidic environment, confirming its stability in acidic conditions, thereby improving the oral delivery of BSA at pH 6.8. Moreover, the increase in BSA penetration across intestinal mucosa further proved the hydrogel’s importance in mucosal delivery of proteins. 

**Figure 7:**
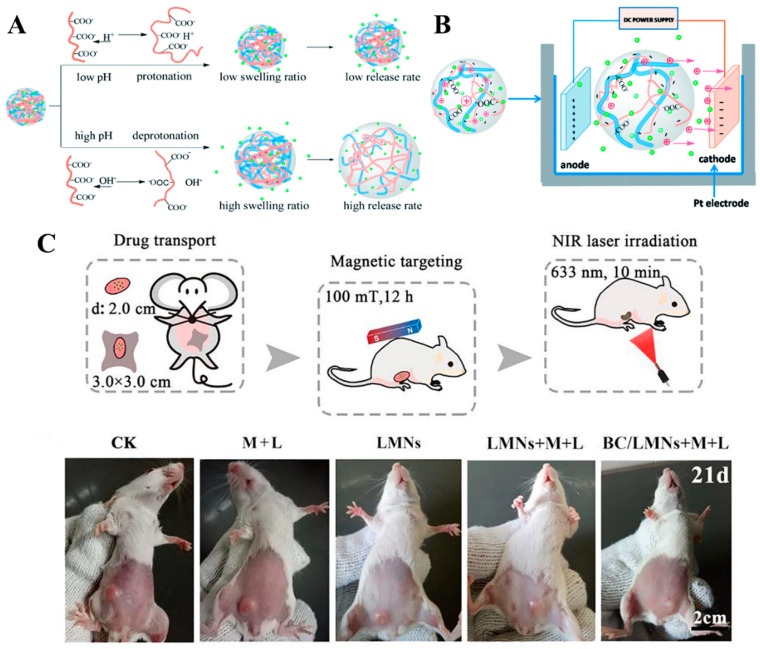
Schematic representation of (**A**) IBU drug release from nf-BC–SA scaffold at different pH conditions, (**B**) under electric field, and (**C**) schematic representation and digital photographs of in vivo efficacy of LMNs treated with tumor-bearing mice by five groups (CK (NS), LMNs, LMNs + M + L, and BC–LMNs + M + L). Reprinted with permission [138,139]. Copyright 2014 Royal Society of Chemistry and 2019 Elsevier.

Shi et al. developed BC–SA hybrid hydrogels with pH and electro-responsive characteristics for the controlled release of IBU [138]. The hydrogels were formed by ionically crosslinking Ca^+2^ with the BC and SA blend. The IBU release depends on the pH swelling of hydrogels. As from Figure 6A, SA showed low swelling at low pH conditions because of protonation of carboxylic groups on SA at acidic conditions. At higher pH conditions, SA could form free carboxylate ions, which promotes the repulsive forces resulted from the higher swelling capacity of hydrogels. In addition to a pH-sensitive property, the hydrogel also responds to the electric field. In the presence of an electric field, the ions could easily be separated, thereby improving the repulsion forces between ions and improving the swelling and drug release from the hydrogel (Figure 7A,B). 

Recently, BC-based materials have been used in cancer treatment applications. Zhang et al. developed BC scaffolds with laser-sensitized magnetic nanoparticles (LMNs) for breast cancer treatment [139]. For effective treatment, doxorubicin and hematoporphyrin monomethyl ether were dually coated on Fe_3_O_4_ magnetic nanoparticles. The LMNs were further conjugated with folic acid for active targeting to cancer cells. The BC scaffolds impregnated with LMNs tested for in vivo chemotherapy and photothermal treatment (Figure 7C). As shown in the figure, showing the in vivo performance of LMNs impregnated in BC, there are no obvious changes in the tumor region of CK (treated with saline only) and M + L groups. The inhibition rates for LMNs, LMNs + M + L, and BC–LMNs + M + L groups were calculated to be 58.85%, 66.87%, and 80.38%, respectively. Therefore, the combination of chemotherapy and photodynamic therapy within the BC–LMNs is promising for breast cancer therapy. The employment of modern technologies, including the immobilization of bioactive agents such as cells and enzymes, has been a demand for mass-produced food. 

#### 2.2.10. BC Scaffolds for Cell–Enzyme Immobilization

The first use of bioactive agents was around 6000 BC or earlier, in baking, the brewing of beer, and the making of wine and cheese, while the first purposeful use of microbial oxidation was around 2000 BC, in the production of vinegar [140,141,142,143]. There has been an evaluation of the application validity in the fermentation industry by using immobilized enzymes altering different parameters in the case of some food items [143,144]. Over the past decade, there has been an emergent interest due to the great potential such as its gelling behavior and high specific surface area in the use of BC as functional materials in bioprocessing technologies, including the delivery system of bioactive agents [27,145]. A number of studies on the delivery and loading of diverse bioactive agents by BC have been reported recently. In 2008, it was established by Wu and Lia that glucoamylase-immobilized BC beads improved the enzyme abilities under changes in the pH and temperature, particularly the lower-temperature section. The activity of the immobilized glucoamylase was 77% at pH 2.0, which was the highest value in the literature up to that time, whereas the activities on their results were above 68% at 20 °C [146]. In 2009, Nguyen et al. reported that *Saccharomyces cerevisiae*, wine yeast, was immobilized into BC through a two-step, adsorption, and incubation for wine fermentation. The immobilizing yeast in BC via a ‘adsorption-incubation’ approach, which was uncomplicated, easy, and low-cost, led to a number of cells in the biocatalyst [147]. There was another study example in winemaking by Ton and Le in 2011. They determined the metabolic activity of *Saccharomyces cerevisiae*, yeast immobilized into BC during repeated batch fermentation for making wine and measured the fermentation performance of the immobilized yeast against the free yeast in a batch fermentation for the reuse process. With 10 cycles of repeated batch fermentation, the immobilized yeast in BC showed better metabolic activities than the free yeast [148]. Montealegre et al. studied the effect of *Saccharomyces cerevisiae*-immobilized Nata de coco on the effluent ethanol concentration, ethanol production rate, conversion, and yield. The results exhibited that the BC biocatalyst accomplishes as good as calcium alginate biocatalyst; moreover, BC’s mechanical stability and cost-effectiveness allow to be an up-and-coming immobilizing matrix for bioethanol production [149]. Kirdponpattara and Phisalaphong fabricated BC–alginate composite sponge through freeze-drying to immobilize yeast cells for ethanol fermentation, with excellent chemical, thermal, and mechanical properties, proper porosity and pore size, and hydrophilicity. It was established that repeat-batch ethanol productions yield using yeast-immobilized BC–alginate composite sponges was more efficient compared to the calcium-alginate matrix or without any matrix. It was considered that efficient ethanol production is due to the water-holding ability and the interconnected porous structures, resulting in appropriate transportation during the process [150]. Similarly, with the study by Wu and Lia in 2008, glucoamylase was also immobilized to BC beads via the periodate oxidation method in 2013. The maximum activity of immobilized glucoamylase enhanced by 40% compared to their previous study was 133.3U/g-BC when glucoamylase was loaded with 1.0% concentration; what is more, it remained by 46% after 14 repeated usages [151]. Tam and Huong reported that the immobilization of Corynebacterium glutamicum in BC via two steps, adsorption and incubation, similarly with Nguyen et al. [147], showed the lysine productivity of 95% in 8 cycles, as well as the survival rate of 80% after 30 days of storage in sterile water, pH 7.0, at 4 °C [152]. Probiotics are live bacteria that provide health benefits for humans, especially the digestive system, increasing in use in the food industry. However, it is poorly transferred from the gastrointestinal system [153]. Fijałkowski et al. reported the potential of BC as an immobilizing matrix of probiotic *Lactobacillus* during co-culture with *G. xylinus*, offering a high-level of protection against gastric juices and bile salts. Moreover, the approach of immobilization with simultaneous cultivation of f *G. xylinus* with *Lactobacillus* in agitated cultures was considered the best way because it allowed almost complete protection of them against gastric juices and bile salts [154]. Khorasani and Shojaosadati designed a BC–pectin nanocomposite with a high prebiotic score and high stability under the gastrointestinal environment, formulated by 43% pectin and 57% BC. The study revealed that BC could be employed as a prebiotic biopolymer to enhance probiotic encapsulation; furthermore, the fabricated nanocomposite enables the stability of *B. coagulans* for long-lived storage at various temperatures [155]. Regrettably, the current studies associated with probiotics-immobilized BC carriers have not focused on evaluating the ability to release probiotic bacteria in the intestine but primarily focused on the impact of technical parameters. In future research, the issue needs to be addressed. Moreover, BC has been widely studied for immobilizing enzymes such as lipase, laccase, and lysozyme, etc., useful to the food industry. Chen et al. conducted a feasibility study on the use of BC as a matrix for immobilizing laccase. It was found that fungal laccase immobilized by crosslinking with glutaraldehyde showed both a broader pH operation range of higher catalytic activity and higher running stability than free enzyme, in which the immobilized laccase was maintained at 69% of the initial activity after seven cycles, resulting in the enzymatic process being economical at an industrial scale [156]. Wu et al. immobilized lipase as a model enzyme into BC membranes via a two-step activation and enzyme attachment. The fixed lipase showed high activity of 93.5% and maintained 60% of the initial activity after 15 cycles [157]. Additionally, Bayazidi et al. immobilized into BC nanofiber through physical absorption method. Their results demonstrated that the pH and temperature of lysozyme activity were extended, and its storage stability was enhanced by immobilizing enzymes [158]. These studies mentioned above induced various BC vehicles, including beads, sponge, paper, and membranes, as a suitable carrier for immobilization of biological agents such as enzymes and cells, which have infinite potentials in food manufacturing.

### 2.3. Paper Industry

The pulp and paper industry are another field with endless potential applications of bacterial cellulose (BC). Since BC has been used as the binder for paper manufacture because it is composed of tiny cellulose microfibril clusters, this property significantly increases the strength and durability of the pulp when used as paper. There is a need for special papers with high strength for archival document repair and long-life currency.

Until now, the paper has been made and distributed in large quantities from woody biomass, which was mainly made by vegetable cellulose. However, since it was first discovered by Adrian J. Brown in 1886 [159,160] that the thin film component produced by acetic acid bacteria in the vinegar-fermenting tablet was cellulose, many studies on the properties of BC have been conducted. This research has led to the focus on the production of BC and its use as paper. Recently, to impart functionality to paper used in various angles, new materials are urgently required, and new functional materials that cannot be obtained from conventional vegetable cellulose using BC are expected. This microbial cellulose has a unique structure and properties such as biodegradability, which is completely degraded in the soil within one month, has high mechanical strength up to several times that of newspaper paper or several times that of aramid fibers, and has a unique structure and properties.

Based on these prior mechanical properties, M. Iguchi et al. [2] found that the addition of degraded BC to wood pulp fibers allowed the creation of a paper sheet with increased tensile strength and folding endurance. Table 1 shows the mechanical propertie sof BC and BC composited papers. The Young’s modulus and tensile strength of composite sheets prepared by filtering the mixture of cotton lint pulp and fragmented BC are measured by Young’s modulus, 4.9 GPa [161]. The folding endurance of pulp papers also significantly improved [162].

D. Johnson et al. [170] proposed improving the tensile properties of paper by using a BC additive. Xiang et al. [167] and Yuan et al. [171] also announced that a homogeneous dispersion of BC within the paper matrix is an important factor to manufacture successfully reinforcing paper. To succeed in this process, intense stirring [172,173] or acid hydrolysis [34] was tried, but the homogeneous dispersal was difficult due to the high entanglement of the nanofibers reached during culture [172]. However, Gao et al. proposed other ways to disperse BC in water before adding it to the pulp. This is made with BC nanofibrillation by a pressure homogenizer and by acid hydrolysis to form a suspension of bacterial nanocrystals. Recently, Campano et al. [174] used soft homogenization to disperse BC in water, forming a gel of nanofibers containing clusters of BC, and they added different proportions to a deinked pulp. 

Asian countries have used Han-ji paper since ancient times, which has many problems to be solved to use it as a printing paper in popular inkjet printers. Han-ji has an inherently rough surface and rapid ink absorption, which significantly reduces bleeding and printability. Kang et al. [175] tried to improve the printability of Hanji using microbial cellulose. They sized Hanji with the BC and various kinds of sizing agents and obtained the results of remarkably improved printability and reduction ink spots. 

#### 2.3.1. Special Functional Paper

BC has DPs of 2000~8000 and is an extracellular cellulose type with a highly swollen (90~95%) state [176,177]. BC is a network structure of ultra-fine and interlaced nanofibers less than 100 nm in width, of which structure provides a large specific surface area and nanoporous structure [167,178]. This nanoporous structure makes it possible to process functionalized BC paper by additionally treating the functional material to the BC support.

Zhang et al. prepared durable fluorescent paper produced from BC–Eu complex, which can form a very stable complex due to BC’s porous structure and shows a great fluorescent property and efficiency [179]. After folding 200 times, the fluorescence intensity of fluorescent paper decreased by only 0.7%, which suggested that the Eu–BC fluorescent paper has great stability and durability. However, it is required for practical use that high production yields and quantitative approaches to these microbial celluloses are insufficient.

BC composite containing gold nanoparticles modified with silica uniform shells (SiO_2_) was manufactured by LBL (layer-by-layer) nanotechnology, which can have special optical properties that appear particularly interesting for security paper applications [180]. 

#### 2.3.2. High-Retaining Water Paper

**Figure 8:**
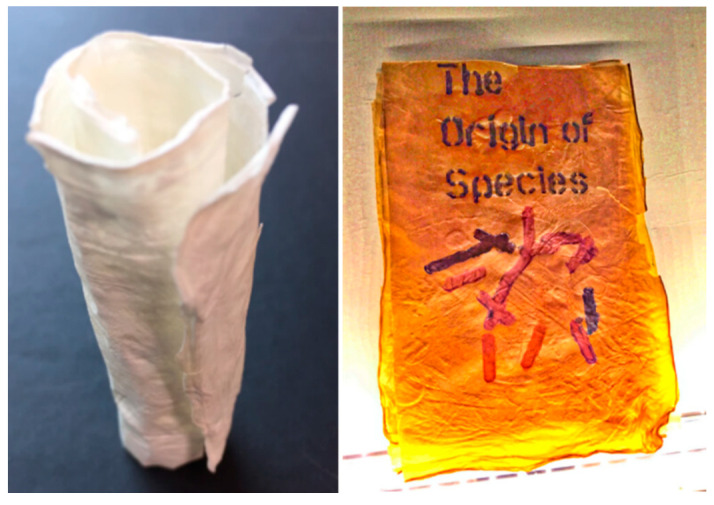
Paper produced with bacterial cellulose [183].

Kang et al. announced that BC from S. ferax has 3.2 (25.04 g/g) times the water-holding capacity and 3.5 (25.75 g/g) times the oil-holding capacity, which were higher than commercial α-cellulose (7.57, 7.25 g/g), respectively [181]. The viscosity of biocellulose is lower than that of commercial α-cellulose. These results indicate the possibility for BC to papermaking application technology with different characteristics from existing wood pulp. However, hydrophilic BC membrane has low permeability to gases in the dry state due to intra- and intermolecular hydrogen bonding networks, which can be changed by water to swell and promotes the collapse of hydrogen bonds, causing a large amorphous region and increasing chain segmental motions. Actually, Marrucho et al. [182] prepared esterified BC membranes that improved the surface and barrier properties by controlled heterogeneous esterification with hexanoyl chloride, which can provide an interesting example in the packaging industry. Several studies have been conducted using BC having such a special function as a raw material for papermaking by Dr. Park [183], as shown in Figure 8, but there are few commercialized examples. 

#### 2.3.3. Packaging Paper 

**Figure 9:**
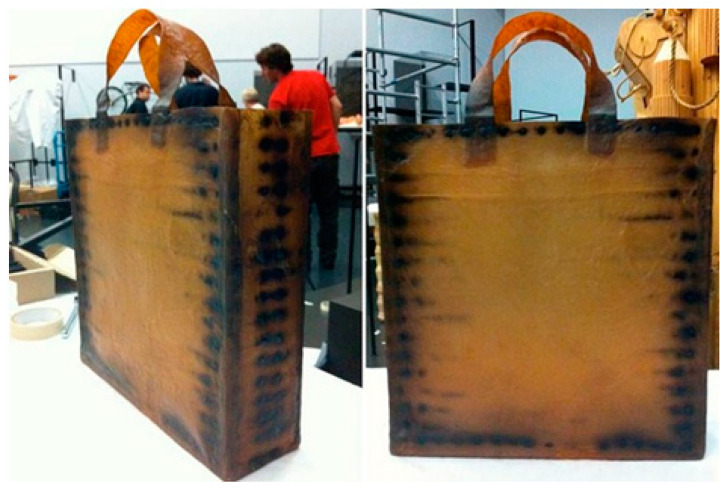
Biodegradable package obtained from bacterial cellulose [184].

Bio packets are packages based on biologically degradable raw materials of plant origin. The plastic bag has become a severe problem in environments, so a lot of research is being conducted on discovering new materials that do not damage the environment. Figure 9 is a biodegradable package obtained from BC, which can have a different service life (on average, from 3 months to 1 year) and a different decomposition period (from 3 months to 3 years), depending on the chosen composition [184]. Bacterial biocellulose is an ecologically clean and biodegradable product. From BC and BC-based composites, they can acquire the packages with outstanding properties including water resistance, superior biodegradability, and mechanical strength. 

**Figure 10:**
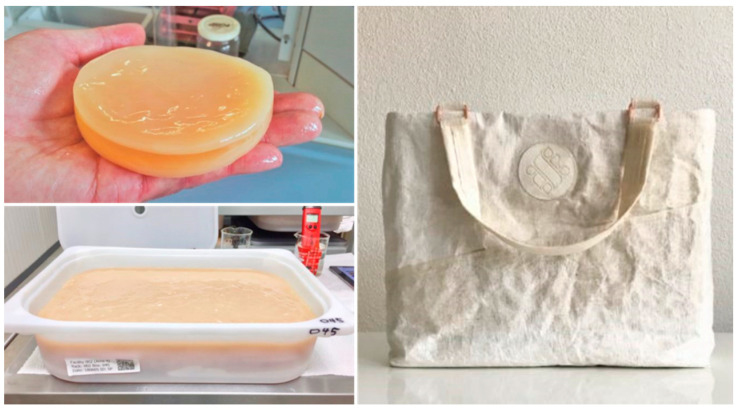
Leather-free handbag made of bacterial cellulose [185].

The material shown in Figure 10 is called ScobyTec BNC, manufactured from Bacterial Nano Cellulose. It is a metabolite of symbiotic bacterial and yeast cultures produced by the fermentation of carbohydrates. This material has high mechanical strength and is non-flammable.

### 2.4. Textile Industry

**Figure 11:**
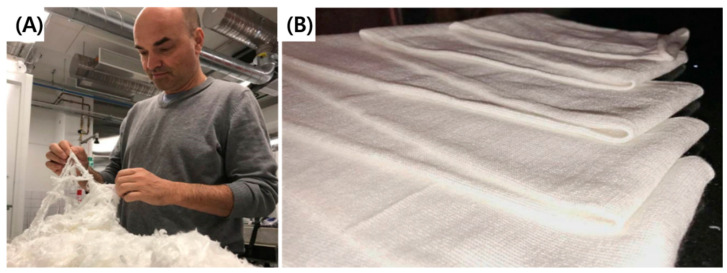
(**A**) Gary Cass, who is now one of the non-executive directors of Nanollose with the Nullarbor Fibre^TM^ and (**B**) fabric made with microbial cellulose [186].

Nanollose (Nedlands, Australia) has developed the world’s first plant-free viscose-rayon fibre manufactured by microbial cellulose, Nullarbor Fibre^TM^ (Nedlands, Australia), which has also been successfully spun into yarn, fabric, and a garment, as shown in Figure 11 [186]. Conventional viscose fibers are made from wood pulp and are used in various ways in clothing and home textiles. However, when making viscose fibers, large amounts of wood must be cut, crushed, treated with harmful chemicals, and then undergo an energy-intensive refining process to obtain the cellulose required for fiber manufacturing. This manufacturing process of regenerated cellulose fiber can have a severe impact on the environment. Nullarbor Fibre^TM^ is manufactured by converting wastes generated from liquid-food industries such as beer and wine into cellulose using microorganisms. 

**Figure 12:**
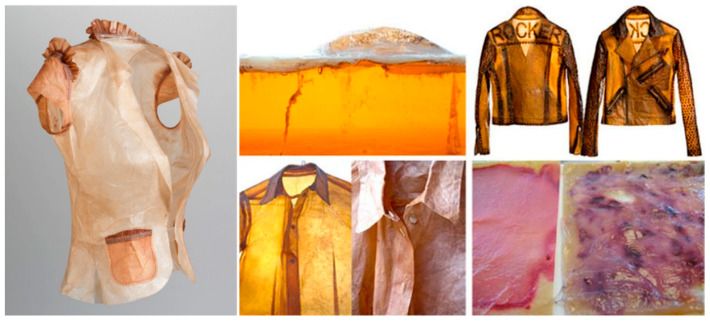
BioCouture using microbial cellulose, grown in a laboratory, to produce clothing [184].

The fashion industry from BC has only recently been announced, which is explored by Suzan Lee [184]. She attempted to cultivate BC sheets for seamless 3D garments directly, which has not been tried, and noted that “The whole thing has been grown and colored in a vat of liquid as opposed to a field of cotton,” referring to the shirts, jackets, and kimonos made by BioCouture (Figure 12). The project’s legacy will likely be its minimal environmental impact rather than fashion credentials. The production of BC requires minimal resources and involves no petrochemicals; the cellulose itself is biodegradable. Although it has grown rapidly with this interest, there are still many technical and real problems associated with BC-based garment manufacturing. 

**Figure 13:**
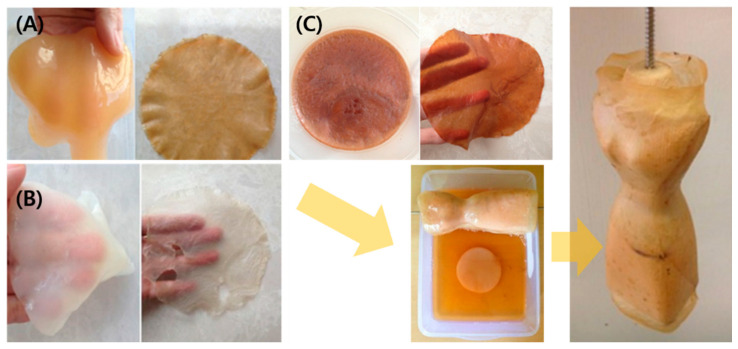
The cellulosic pellicles grown from the selected types of BC (**A**) beer, (**B**) milk, and (**C**) wine. Reprinted with permission from [187]. Copyright 2016 Taylor & Francis.

Wang et al. [187] investigated various types of BC, such as beer, milk, green tea, black tea, and red wine, and suggested that cellulose grown from green tea was identified as the most effective for fashion creation, as shown in Figure 13. Furthermore, they successfully manufactured 3D self-grown directly from BC with or without embellishment and ornamentation.

**Figure 14:**
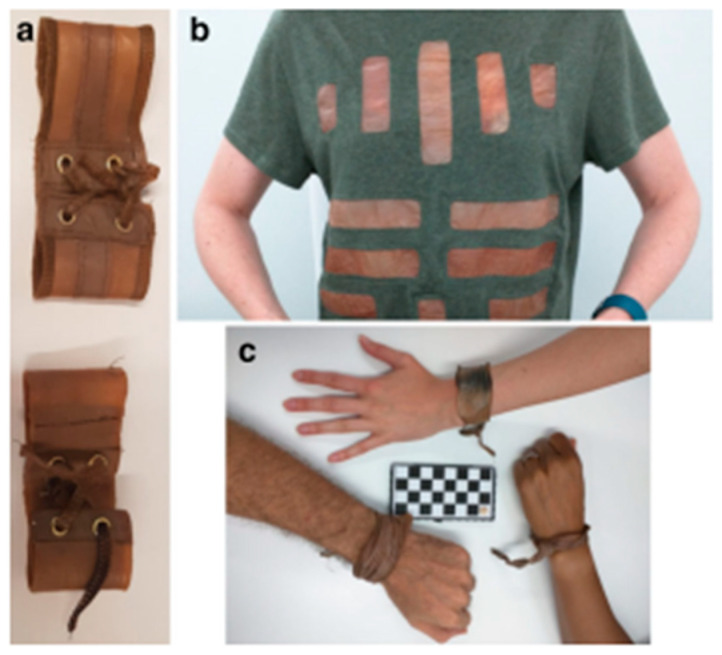
Photographs of the finished articles of clothing: (**a**) wristband, (**b**) t-shirt with sewn in cellulose-based coated fabric, and (**c**) wristband after 2 weeks of use. Reprinted with permission from [168]. Copyright 2020 Springer Nature.

Recently, Kamil Kamiński et al. proved that BC is adequate to manufacture various parts of clothing using common sewing techniques [188]. They tested them on volunteers to determine the skin-to-skin contact behavior of the BC clothes (Figure 14). The use of glycerol with BC by introducing it to the structure as a water-retaining agent and transforming it into a stable hydrogel improved the mechanical properties of the finished fabric. It also demonstrated a procedure for synthesizing an almost ready-to-use fabric from raw materials.

### 2.5. Electrical and Electronic Industries

Recently, as electronic devices have become highly densified and miniaturized, the need for structures with ultra-fine patterns at the nano level is increasing. Carbon nanomaterials such as carbon nanotubes and nanofibers are attracting attention because of their high specific surface area, electrical conductivity, and mechanical strength for electronic devices. Several methods have been tried for continuous synthesis for mass production of carbon nanomaterials, but satisfactory results have not been obtained. However, Kim et al. [188] confirmed that the formation of tar was reduced by heat treatment of BC in HCl vapor before the carbonization process and yield fibrous structure of the carbonized sample. 

The porous, bulk BC materials can expand their applications in electronic devices with properties unlike dense materials such as metal or conductive film [189]. Studies have been conducted to improve conductivity through the polymerization of conductive polymers on the surface of BC. As a result, the conductive polymer can be polymerized on the surface of the modified BC three-dimensional network structure by the silane pretreatment. It has been proven that the conductivity is improved. The prepared conductive polymer–BC composite material is expected to be applicable to biological sensors, low-capacity energy devices, biocompatibility, and electrical conductivity [190,191]. Research to manufacture an electrically conductive material using BC as a binder or support is one of the most active fields. The chemical structure with hydroxyl and ether group of BC provides an excellent hydrophilic matrix for nanoparticle incorporation. For example, there are several articles using carbon nanotubes (CNT) that endue conductivity electrically to BC. However, it is difficult to disperse CNTs due to forming stabilized bundles through van der Waals forces. On the other hand, the BC is much more flexible than using ordinary composite fabrication. The method of CNT use is divided into: one is immersing BC pellicles in multiwall (MW) CNTs dispersion with a surfactant, which is generally used to improve the dispersion of carbon nanotubes in an aqueous solution. This is a complex process of electrically conducting cellulose pellicles containing well-dispersed and embedded MWCNTs [192]. The other is harvesting BC membranes in a CNT-dispersed solution using amphiphilic comb-like polymer (CLP), which has no toxicity and is used to maintain the activity of bacteria. That is, harvesting BC with electricity by the one-step biosynthesizing process shows higher conductivity and smoother morphology [193]. 

BC complexed with graphene oxide has improved battery conductivity and mechanical strength, which has been evaluated as a material applicable to biochemical and electrochemical devices [194]. Research about imparting conductivity to BC using a conductive polymer has been actively published. Polyaniline and polypyrrole are synthesized with nanoparticles on the surface of nanofibers constituting BC pellicle to prepare an electrically conductive three-dimensional structure. In particular, a magnetic nanoparticle cluster prepared by introducing a surfactant was introduced into a bacterial culture solution to form a magnetic cellulose pellicle. Park et al. [194] biosynthesized magnetic BC by culturing bacteria in a medium containing well-dispersed magnetic particles (MNPs), and then synthesized polyaniline on the surface of BC–MNPs composites fibers as polymerization sites. The results show that polyaniline nanoparticles form 3D networks, and the composite film is electromagnetic and can be a potential shielding material. They also manufactured a BC–silicon-nanoparticles–polyaniline composite for an anode material for Li-ion rechargeable batteries [195]. Kim et al. [196] present the enhancement of the electrical conductivity and washing durability of a BC–polyaniline membrane by in situ synthesis using the addition of metal salt. The results show that the addition of 0.05% (*w*/*v*) of copper (II) sulfate in BC–PANI presents 7.54 × 10^−2^ S/cm electrical conductivity.

**Figure 15:**
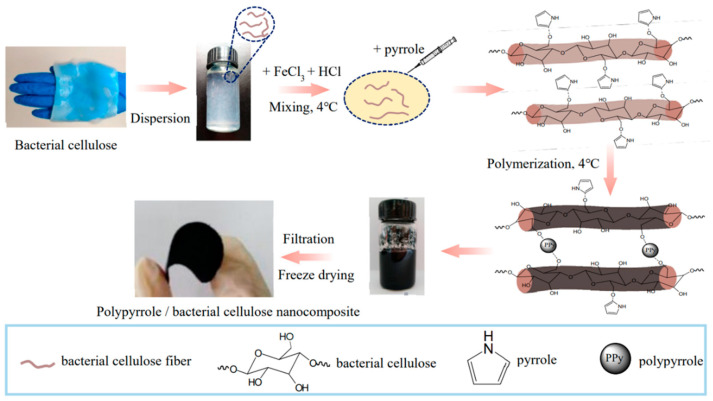
Schematic illustration of preparation PPY–BC nanocomposite. Reprinted with permission from [198]. Copyright 2019 MDPI.

Polypyrrole(PPy)–BC nanocomposites have been studied extensively for flexibility and for supercapacitors. Recently, these materials promoted the rapid development of intelligent and wearable textiles. However, aggregation of PPy on the BC surface has been a problem, so the electrical conductivities of the composites were relatively low 2.1~6.8 S cm^−1^. To improve this material, a flexible and conductive nanocomposite biocellulose film was prepared by in situ chemical polymerization of pyrrole using BC as a three-dimensional template, as shown in Figure 15 [197]. In this report, the experimentally determined response of (7.34 ± 0.25) S cm^−1^ was reasonably apparent. Moreover, the composite exhibited a high areal capacitance of 1001.26 mF cm^−2^ at the discharge current density of 1 mA cm^−2^, but its cycling stability could be further improved. 

This can be applied to a shielding film to block harmful electromagnetic waves, and research on this is ongoing. These results are summarized in Table 2. 

#### 2.5.1. Electrical Display Device

**Figure 16:**
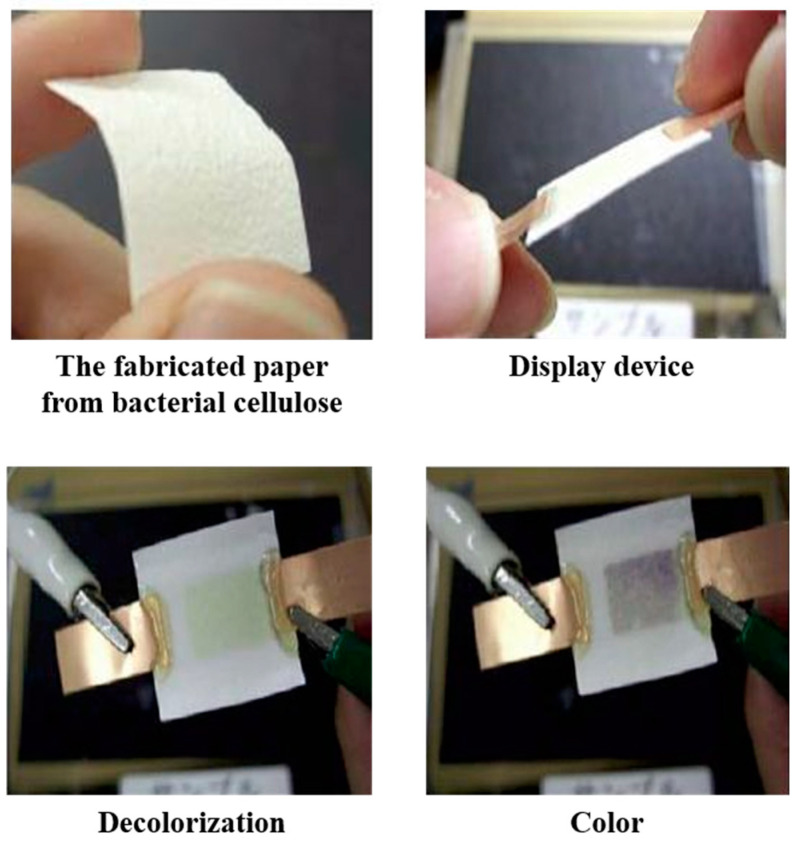
Electrodes directly on paper made from BC [203].

Japan’s Ricoh, Agri Bio-Industry, and Tajima et al. of the Graduate School of Engineering at Hokkaido University have formed electrodes directly on paper made from BC and then colored by penetrating the pigment into the paper, as shown in Figure 16. The display color test was successful. It does not require a supporting base (glass windows or plastics) essential for a conventional display. Since paper can be colored or removed, it is possible at a low cost due to the small number of subsidiary materials and simple manufacturing processes [203].

At the same time, it is attracting attention as a green technology that is harmless to the environment because it uses a low-energy manufacturing process by bacteria without using forest resources. A display device has been developed using bendable BC as a substrate and developing a cellulose display combined with depositing ions, and it is expanding its utility to electronic newspapers, e-books, and dynamic wallpaper. Cultured BC has a stable dimension, paper-like appearance, and unique microfiber nanostructures to achieve electronic display. To provide a conduction path, ions are deposited around the microfibrils, and then electrochromic dyes are immobilized within the microstructure of the BC sheet. Then, the whole system is cased between the transparent electrodes, and the switching potential (2–5 V) can be reversible. The color variations can be demonstrated up to the standard pixel-sized areas (about 100 μm^2^). The main advantages of these devices are their paper-like high reflectivity, flexibility, contrast, and biodegradability [204]. 

**Figure 17:**
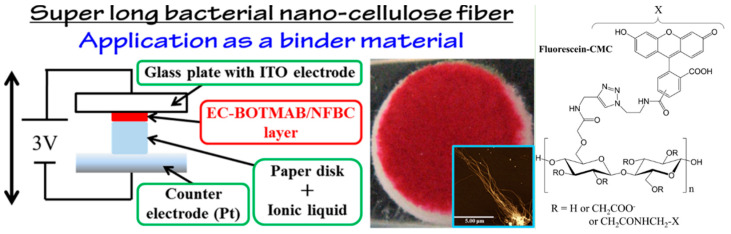
Experimental equipment for the coloring test and images of the EC-BOTMAB/NFBC. Reprinted with permission from [205]. Copyright 2019 American Chemical Society.

Recently, Kenji Tajima et al. prepared a nanofibril bacterial cellulose (NFBC) production system via a bottom-up process with carboxymethyl cellulose (CMC) as a dispersing agent, as shown in Figure 17. They used this NFBD as a binder for a display device by using an electrochromic (EC) material, which shows a reversible color change under an electric field. They introduced quaternary ammonium into EC molecules for enhancing interactions with NFBC and observed a successful homogeneous color change [205]. 

#### 2.5.2. OLED

Flexible and transparent composites of BC can be extended to manufacture organic light-emitting diodes (flexible OLEDs). OLED substrates composed of cellulose and polymer have been reported. Legnani et al. [206] prepared an OLED substrate from a BC sheet deposited with SiO2. The OLED circuit was fabricated by radio frequency magnetron sputtering high-conduction ITO into BC flexible membrane with and without a SiO_2_ interlayer. The maximum luminance of this material was 1200 cd/m^2^, which shows a possibility to develop biocompatible OLED devices. Since then, research on transparent BC nanocomposites film as a substrate for flexible OLED has continued. Manuspiya et al. [207] used BC and polyurethane (PU) as a substrate for OLED display. The visible-light transmittance of the nanocomposite film was as high as 80%, using PU with a similar refractive index close to that of BC. Its thermal stability was stable up to 150 °C. After that, OLED was successfully fabricated on the nanocomposite via thermal evaporation deposition. Messaddeq et al. [208] also fabricated flexible and transparent freestanding films with BC–PU composites for lighting-emitting devices and displays, which have high transparency in the ultraviolet (up to 70% at 350 nm) and visible regions (up to 90% at 700 nm). For the application of flexible OLED, BC–PU film was coated with SiO_2_ and indium tin oxide (ITO) thin film, and luminance results of the flexible OLED were 231 ± 18 cdm^−2^. Recently, Legnani et al. [209] used high-transparency BC as a substrate. They also manufactured BC–boehmite (Boe) nanoparticles epoxy-modified siloxane (GTPS) membranes covered with silicon dioxide (SiO_2_) and indium tin oxide (ITO) in OLED. The maximum efficiencies for BC/SiO_2_/ITO substrate was 1.68 cd/A. These results suggest that BC nanocomposites are an exciting new path to the eco-friendly biocompatible substrate for flexible electronic applications. 

#### 2.5.3. Fuel Cell

BC for the design of biosensors and biofuel cells are also being actively developed. The BC possesses reducing groups capable of initiating the precipitation of metals, such as palladium, from aqueous solution and suitable for the construction of membrane electrode assemblies (MEAs), which has the ability to catalyze the generation of hydrogen when containing sodium dithionite and generated an electrical current [210]. Recently, research on BC as an anode material for Li-ion rechargeable batteries has been published. Park et al. [195] describe a conductive BC composite with silicon nanoparticles and polyaniline. Hydroxyl groups by phytic acid treatment were introduced on the surface of silicon nanoparticles (SiNPs), and hydrogen bonds were formed with BC nanofibers. After adding an aniline monomer and APS, PANi was polymerized on both BC nanofibers and SiNPs. The BC structure can serve as the template for both the adsorption of SiNPs and the polymerization of PANi. The phytic acid introduced on the surface of the silicon particles has the advantage of increasing the surface adhesion efficiency of the conductive polymer by forming a hydrogen bond with the aniline monomer.

#### 2.5.4. Flexible Supercapacitor

**Figure 18:**
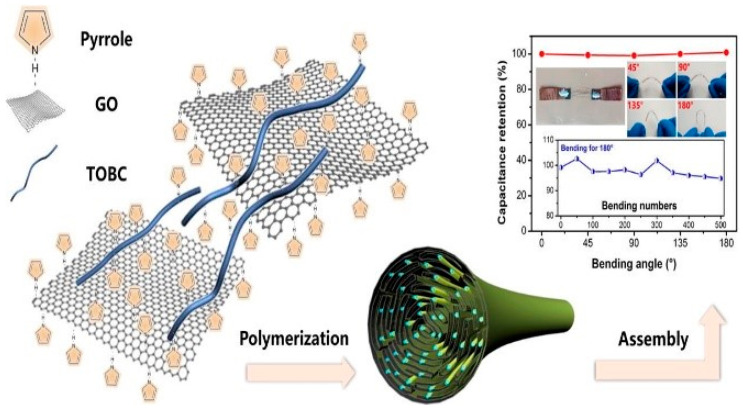
Flexible supercapacitor based on the polypyrrole@TEMPO-oxidized bacterial cellulose–reduced graphene oxide (PPy@TOBC–rGO) macrofibers. Reprinted with permission from [219]. Copyright 2019 Elsevier.

A flexible supercapacitor (SCs) based on BC has been developed. Commercial SCs are mainly based on rigid batteries, and the demand for flexibility continues. Replacing rigid electrodes with flexible or stretchable electrodes will be the development trend of SCs in the future. BC is a promising flexible material with a high aspect ratio, high water retention capacity, excellent mechanical strength, and low cost [211,212,213]. Cellulose nanofibers contain electrolyte absorption properties and can diffuse them into energy storage materials while providing good diffusion channels for electrolyte solution and enhancing ion transportation to the active materials [214,215,216,217,218,219,220] Recently, Wang et al. [219] developed a facile and scalable method for the preparation of flexible, binder-free high performance all-solid-state flexible SCs based on the polypyrrole@TEMPO-oxidized BC–reduced graphene oxide (PPy@TOBC–rGO) microfibers, which exhibits a high energy density of 8.8 mWh cm^−3^ at the power density of 49.2 mW cm^−3^, which is better than most previously reported graphene flexible SCs, as shown in Figure 18. In addition, the high electrochemical performance of these SCs is maintained after cycling and bending.

These SCs are excellent in mechanical properties, chemical stability, resistance to bending, and durability. This study shows that the field of a new material called BC could be the fundamental basis for the development of flexible SCs in the future.

#### 2.5.5. Stereo Headphones and Monitors

BC can be used for audio speaker diaphragm products. In general, the sound wave velocity is proportional to the square root of the specific tensile modulus, which is the value obtained by dividing the tensile modulus by the density. However, BC has a high loss coefficient despite its high sound wave speed (5000 m/s) and is used in the diaphragm of speakers and headphones, which are acoustic devices [220]. The acoustic membrane using a low compressed thickness (~20 µm) of high-end headphones (VSonic GR-07 iem: VSonic used nearly 100 very thin layers of biocellulose membranes) made of BC is manufactured by Sony [161]. In order to be used in this application, characteristics such as high Young’s modulus value, sound propagation speed, and relatively high internal loss are required. The diaphragm of early loudspeakers was made of paper (so-called cone paper) because it can be processed into a light diaphragm. The inherent dimensional stability of microbial-made cellulose makes it the best material for optimal sound conversion by creating a membrane that can maintain higher sound speeds over a wide frequency range. Moreover, it is reported that BC exhibits higher modulus values and higher internal loss values compared to diaphragms made of cone paper.

**Figure 19:**
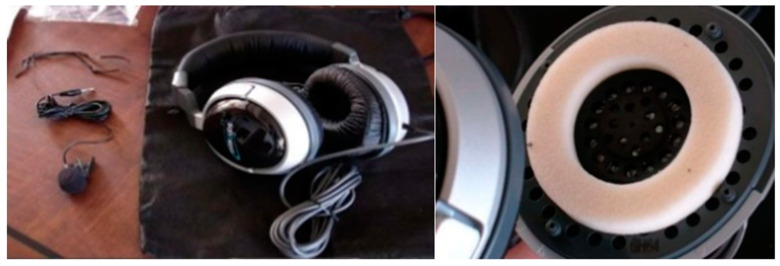
Biocellulose as membrane transducer in Sony headphones [221].

At present, Sony produces more than KRW 100,000 trillion of high-quality speakers a year, significantly improving the reproduction of bass by using BC as the acoustic diaphragm for speakers in Figure 19. The acoustic diaphragm must have a high sonic velocity and a high internal loss, which is a property of internally absorbing sound waves.

#### 2.5.6. Electromagnetic Wave Absorbing Materials

**Figure 20:**
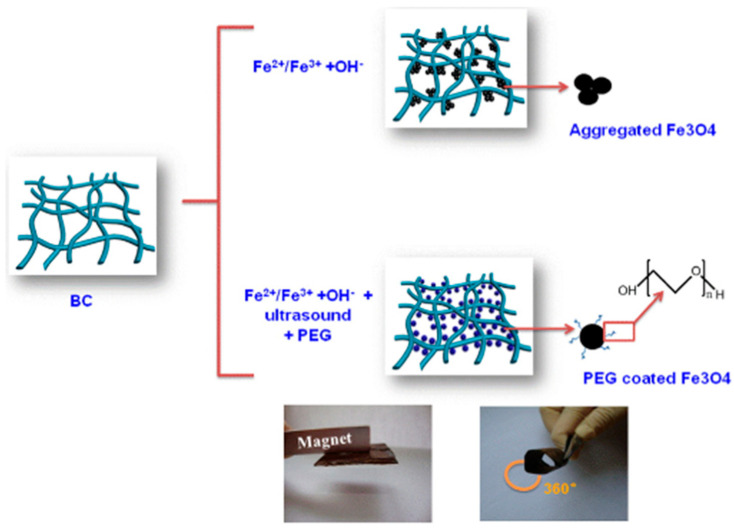
Schematic illustration for the Fe_3_O_4_–BC matrix process. Reprinted with permission from [225]. Copyright 2013 Elsevier.

The properties of BC as a nanoparticle-absorbing matrix include having a large number of sub-micron pores, which helps to stabilize the precipitated nanoparticles for the in-situ magnetite synthesis and provides the possibility for the formation of nanoparticles due to the hydroxyl group as active sites for the metal adsorption. Moreover, ultrafine network architecture with a distinct tunnel, pore structure, and higher specific surface area with a great deal of hydroxyl groups helps stabilize the precipitated nanoparticles [222,223,224]. A BC complex containing Fe_3_O_4_ magnetic nanoparticles with chemical stability and excellent magnetic properties was synthesized as an electromagnetic wave absorbing material. The magnetic nanoparticle complex prepared in this way can be applied to fields such as drug delivery, bioseparation process, magnetic resonance imaging, and magnetic fluids, so it is a material that can be widely used in disease diagnosis and treatment and biotechnology. Zheng et al. [225] fabricated a magnetic BC membrane through the in situ synthesis of Fe_3_O_4_ nanoparticles coated with PEG into BC matrix under ultrasonic irradiation, as shown in Figure 20. Marins et al. [226] also presented a BC–Fe_3_O_4_ magnetic nanoparticles composite, which also deals with the effect of the magnetite nanoparticle on the electromagnetic interference (EMI), shielding the effectiveness of the corresponding BC-based magnetic membranes. The coercive force of these nanoparticles stayed in the range of 15 Oe, which indicates superparamagnetic behavior. Therefore, this material could be used for nonlinear optics, and could have clinical applications as a contrast agent for magnetic resonance imaging, hyperthermia, cell separation, and sensors.

### 2.6. Etc.

Using the unique BC nano-porous 3D network structure and high specific surface area endowing the surface of micro-fiber with a great deal of hydroxyl and ether bonds, Hu et al. [227] synthesized ZnO nanoparticles and nanowires on BC as a template or matrix. By varying the zinc acetate aqueous solution concentration, zinc oxide nanoparticles with a controlled particle size and morphology were prepared. The calcinating at 600 °C with 1 wt % zinc acetate aqueous solution is adequate to obtain well-dispersed spherical ZnO nanoparticles with a narrow size distribution and high photocatalytic activity. Additionally, BC nanofibrous composites containing TiO_2_ were biosynthesized with a high specific surface area of 208.17 m^2^/g and an average particle diameter of 4.3~8.5 nm, which shows high photocatalytic properties [228]. The mechanism of arraying spherical TiO_2_ nanoparticles involves using BC nanofibers to enhance photocatalytic activity with a high specific surface area [229,230]. Therefore, this approach provides an environmentally friendly and efficient technique for the preparation of the well-dispersed nanoparticles. This method could also be used to prepare nanoscaled metal oxide from the thermal decomposition process.

Recently, CMC (carboxymethyl cellulose) and BC complex were prepared for surfactant by the spray-drying method. The prepared composite has a zeta potential of −67.0 ± 3.9 mV and a particle diameter of 601 ± 19.7 μm. It has excellent characteristics such as reduced interfacial tension, high viscosity, 3D network formation ability, and surface charge. It showed the possibility of being a stabilizer in oil-in-water emulsions [231]. 

## 3. Concluding and Future Trends

In the context of pursuing sustainable eco-materials, BC must be one of the best materials to meet the latest trends due to its inherent and unique properties, including biocompatibility, non-toxicity, biodegradability, gas permeability, suspension stability, low viscosity, high water-holding capacity, and tolerance to acid/salt/ethanol. These are also recyclable, environmentally friendly, and could create alternatives to petroleum-based materials. While it is appropriate for a wide range of applications in industrial and research, the limitations of high manufacturing cost and low yields could not be overcome for the large-scale production of BC and its commercial applications. Accordingly, further endeavors have still been required in order to improve the mentioned issues related to the production efficiency, the yield of BC, and new cost-effective approaches. The static fermentation has limited production capacity due to its labor-intensive and time-consuming process, and the agitated fermentation reduces the yield of BC owing to non-cellulosic mutations of bacteria, despite being able to produce BC on a large scale. Accordingly, it is necessary to improve the production efficiency and yield of BC through variable approaches, such as high-yield isolation of BC strains, developments of new culture media and reactor, and use of automated equipment. Additionally, its cost is expensive compared to plant cellulose, and industrial waste including coconut water is becoming more popular with growing marketing requirements. In line with this situation, a new cost-effective source of nutrition, for example, liquid wastewater or fruit juice, could be used for BC production. The related mechanisms should be clearly established in order to provide a theoretical foundation for exploring novel functionality in versatile industries. Therefore, in-depth discussions are needed to focus on key advances for the next few years. Taking advantages of BC will maintain competition in the market.

## Figures and Tables

**Table 1:** The mechanical properties of bacterial cellulose (BC) and BC composited papers.

Method	Tensile Strength (MPa)	Young’s Modulus (GPa)	Folding Endurance (Times)	Ref
Mixture of cladophora and fragmented BC	35~50	2.3~4.0	10~58	[162]
BC nanofibers–polyester composites	18~22	0.58~0.72	-	[163]
BC nanowhisker-reinforced polylactic acid	25~27	1.1~1.3	-	[164]
BC–polyaniline nanocomposite paper	-	-	13~50	[165]
Pulp-reinforced BC	0.1~0.15 (burst strength)	-	-	[166]
BC addition to pulp paper	>79.9 N m/g (tensile index) >5 kPa m^2^/g (burst index)	-	-	[167]
Raw BC	5	0.04		[168]
Hydrogel BC with glycerol	8	0.28		[168]
BC film obtained through Kombucha	0.4~12.8 (different to dry states)	0.02~2.65		[169]
Mixture of cotton lint and fragmented BC	1~100	0.1~4.9	-	[164]

**Table 2:** Electrical properties of BC composites.

Method	Electrical Conductivity (S/cm)	Properties	Ref
LBL multilayering of polyethylene imine(PEI) and poly(3,4-ethylene dioxythiophene):poly(styrenesulfonate) (PEDOT:PSS)	10^−5^~10^−4^	Without loss of paper strength	[189]
multiwalled carbon nanotubes (MWCNTs) into BC	0.14	Purity, high crystallinity, ultrafine network	[192]
BC is cultured in a carbon nanotube (CNT)	0.104	High CNT stability by the one-step biosynthesizing	[193]
BC is cultured in a medium containing magnetite nanoparticle (MNP) clusters	0.43	BC fibers was fully coated with polyaniline, forming hydrogen bonds.	[194]
BC composite with silicon nanoparticles(SiNPs) and polyaniline.	0.017	Anode material for Li-ion rechargeable batteries	[195]
BC–polyaniline (PANI) membrane by the addition of metal salt.	0.075	washing durability is improved	[196]
Pyrolyzed BC–polydimethylsiloxane	0.2~0.41	High tensile and bending strain	[197]
polypyrrole nanocomposite membranes based on BC	0.32	good electromagnetic shielding effectiveness	[199]
wrapping a homogenous layer of polypyrrole (PPy) around BC nanofibers	77	for supercapacitors, with a highest mass-specific capacitance hitting 316 F/g at 0.2 A/g current density.	[200]
in situ oxidative polymerization of pyrrole (Py) in the presence of BC membrane	0.01–1.2	good mechanical properties (40 MPa)	[201]
in situ chemical polymerization of polypyrrole–BC	7.34	a core-sheath structure exhibited higher thermal stability and flexible	[198]
BC–GO nanocomposite using vacuum-assisted self-assembly technique	1.1	Well-dispersed GO nanosheets in the BC matrix, flexible and approved mechanical film	[202]
BC–polyaniline nanocomposite film by chemical oxidative polymerization	1.3	BC fibers were fully encapsulated by polyaniline spherical spheres	[190]

## Data Availability

Not applicable.
